# Spotlight on nuclear PD-L1 in ovarian cancer chemoresistance: hidden but mighty

**DOI:** 10.3389/fimmu.2025.1543529

**Published:** 2025-07-14

**Authors:** Meshach Asare-Werehene, Arvin Zaker, Shivanshi Tripathi, Laudine Communal, Euridice Carmona, Anne-Marie Mes-Masson, Benjamin K. Tsang, Arvind Mer

**Affiliations:** ^1^ Department of Obstetrics & Gynecology, University of Ottawa, Ottawa, ON, Canada; ^2^ Department of Cellular and Molecular Medicine & The Centre for Infection, Immunity and Inflammation (CI3), Faculty of Medicine, University of Ottawa, Ottawa, ON, Canada; ^3^ Inflammation and Chronic Disease Program, Ottawa Hospital Research Institute, Ottawa, ON, Canada; ^4^ Department of Laboratory Medicine and Pathobiology, University of Toronto, Ottawa, ON, Canada; ^5^ Department of Biochemistry, Immunology and Microbiology, University of Ottawa, Ottawa, ON, Canada; ^6^ Centre de recherche du CHUM et Institut du cancer de Montréal, Département de médicine, Université de Montréal, Montréal, QC, Canada; ^7^ School of Electrical Engineering and Computer Science, University of Ottawa, Ottawa, ON, Canada; ^8^ Ottawa Institute of Systems Biology, University of Ottawa, Ottawa, ON, Canada

**Keywords:** PD-L1, immunotherapy, nuclear PD-L1, ovarian cancer, chemoresistance, tumor microenvironment, plasma gelsolin (pGSN)

## Abstract

**Introduction:**

Ovarian cancer (OVCA) has a five-year survival rate of approximately 45%, with little improvement over recent decades. Although anti-PD-L1 therapies have shown substantial efficacy in other solid tumors, their effectiveness in OVCA has been limited. These treatments target only membranous and soluble forms of PD-L1, without addressing nuclear-localized PD-L1. The role of nuclear PD-L1 in OVCA chemoresistance, however, remains largely unexplored. In this study, we examined the prognostic significance of nuclear PD-L1 and its interactions with plasma gelsolin (pGSN) and CD8+ T cells within the tumor microenvironment.

**Methods:**

Using immunofluorescence, we quantified nuclear PD-L1, pGSN, and additional markers in OVCA samples. Statistical analyses and machine learning approaches were employed to assess associations between marker expression, patient outcomes, and chemoresistance.

**Results:**

Increased nuclear PD-L1 was associated with disease recurrence, chemoresistance and poor overall survival. Although CD8+ T cells provided survival benefits to patients, elevated PD-L1 hindered these benefits resulting in shortened disease free (DFS) and overall survival (OS). Co-expression of PD-L1 and pGSN was also associated with shortened DFS, OS and chemoresistance.

**Discussion:**

These findings indicate that nuclear PD-L1 serves as a poor prognostic marker in OVCA, being associated with tumor recurrence, chemoresistance, and reduced overall survival. Targeting nuclear PD-L1 may represent a novel therapeutic strategy to improve outcomes in patients with OVCA.

## Introduction

Ovarian cancer (OVCA) is a lethal gynecological malignancy with a 5-year survival rate of ~45% (1, 2). The first line treatment is surgical debulking coupled with chemotherapeutic agent. Complete cytoreductive surgery (CCR 0), where no macroscopic disease remains, is strongly associated with improved overall survival. In contrast, patients left with residual macroscopic disease (CCR 1–3) after surgery face significantly poorer outcomes. Notably, the potential for cure in cases with substantial residual tumor burden at the conclusion of surgery is exceedingly limited. These distinctions underscore the importance of optimal surgical planning and execution in improving patient prognosis. Although patients initially respond to treatment, late diagnosis and chemoresistance present as major obstacle to treatment success.

Treatment response in OVCA is modulated by the tumor microenvironment especially the immune system which plays a crucial role in the recognition and elimination of tumor cells. Interestingly, OVCA cells can evade immune surveillance by expressing immuno-suppressive molecules such as pGSN and PD-L1 (3–7). pGSN is highly expressed in OVCA tissues and activates the Akt/HIF1alpha axis resulting in chemoresistance (8). Increased expression of pGSN by OVCA tissues inhibits the anti-tumor functions of CD8+ T cells, dendritic cells, NK cells and macrophages in the tumor microenvironment leading to tumor recurrence, chemoresistance and poor survival (9–12).

PD-L1 binds to its receptor, programmed death 1 (PD1), on the surface of T cells and inhibits their activation and function, resulting in reduced T cell cytotoxicity and increased tumor growth (13–15). Previous studies have shown conflicting results regarding the prognostic value of PD-L1 in the ovarian tumor microenvironment (15). A study by Høgdall et al., revealed that PD-L1 over-expression was observed in ~50% of all OVCA cases and associated with advanced stage and tumor aggressiveness (16). Meanwhile other studies have shown a positive correlation between PD-L1 expression and T cell infiltration suggesting PD-L1 expression as a potential predictive biomarker for immunotherapy (17–19). Investigation into the underlying functional dynamics of PD-L1 is crucial for developing highly effective and personalized therapies to achieve optimal responses in ovarian cancer patients. Targeting the PD-L1/PD1 axis is a promising strategy to enhance antitumor immunity and improve clinical outcomes in many malignancies. Despite the significant treatment responses achieved in other solid cancers, anti-PD-L1 inhibitors have produced only modest therapeutic success in OVCA patients (20–23). These conventional antibodies only target membranal and soluble PD-L1 without any effect on intracellular PD-L1. Nuclear PD-L1 has recently been detected in a variety of malignancies including breast cancer, colon cancer, lung cancer and prostate cancers (23–27). Although their function in the nucleus is not well established, it’s been shown to induce transcription of genes leading to angiogenesis, tumorigenesis, drug resistance and tumor recurrence (26–28). It is currently unknown if nuclear PD-L1 is detectable in OVCA tissues. Whether its presence in OVCA tissue might explain the modest therapeutic responses seen with anti-PD-L1 immunotherapies is not known.

In this study, we report for the first time the detection and prognostic value of PD-L1 expression in the nucleus and cytoplasm of OVCA tissues. We also provide findings on the relationship between nuclear PD-L1, pGSN and T cells and how their interaction correlates with tumor recurrence, chemoresistance and patient survival.

## Materials and methods

### Ethics statement and tissue sampling

The study was in accordance with the appropriate guidelines approved by the Centre hospitalier de l′Université de Montreal (CHUM) Ethics Committee [Montreal, Quebec, Canada, Institutional Review Board (IRB) approval number, BD 04-002] and the Ottawa Health Science Network Research Ethics Board (Ottawa, Ontario, Canada, IRB approval number, OHSN-REB 1999540-01H). 208 subjects enrolled in the study were treatment naïve patients with OVCA from 1992–2012 at the CHUM (Montreal, Quebec, Canada) and provided written informed consent. Thus, these patients received no radiotherapy nor neoadjuvant chemotherapy prior to sample collection at surgery. Tissue microarrays (TMA) were built with a total of 208 formalin-fixed OVCA tissue samples, 14 normal fallopian tube samples and cell line pellets (TOV122D and OV2295) in duplicates. Details of patient population is described in **Table 1**. BRCA status was unavailable for a subset of samples, resulting in a total count lower than the full cohort (n=208) in **Table 1**. Patients were diagnosed, tissues examined, and clinical data gathered as described previously (9).

**Table 1:** Clinical characteristics of the patients used in the analysis.

Variables	All	BRCA non-carriers	BRCA1 carriers	BRCA2 carriers	P
N	208	127	14	17	
Age (mean ± SD)	61.75 ± 10.26	63.14 ± 9.09	51.43 ± 10.48	58.00 ± 8.51	<0.001***
Menopause Status (%)					
Postmenopausal	142 (90.4%)	90 (93.8%)	8 (72.7%)	11 (84.6%)	0.052
Premenopausal	15 (9.6%)	6 (6.2%)	3 (27.3%)	2 (15.4%)	
FIGO Stage (%)					
Stage 1	13 (6.3%)	8 (6.4 %)	1 (7.1%)	2 (11.8%)	0.042*
Stage 2	20 (9.6%)	8 (6.4 %)	4 (28.6%)	2 (11.8%)	
Stage 3	153 (73.6%)	96 (76.8%)	9 (64.3%)	9 (52.9%)	
Stage 4	22 (10.6%)	13 (10.4%)	0 (0.0%)	4 (23.5%)	
CA 125 (mean ± SD)	1128.5 ± 1768.7	1246.4 ± 2019.7	1576.4 ± 1924.7	1053.3 ± 848.2	0.752
Residual Disease (%)					
Optimal	86 (44.1%)	51 (44.0%)	7 (53.8%)	8 (50.0%)	0.739
Suboptimal	109 (55.9%)	65 (56.0%)	6 (46.2%)	8 (50.0%)	
Histopathology (%)					
HGS	174 (81.7%)	104 (97.2%)	13 (92.9%)	16 (100.0%)	0.651
LGS	5 (2.3%)	3 (2.4%)	0 (0%)	0 (0%)	
Endometrioid	1 (0.5%)	0 (0%)	0 (0%)	0 (0%)	
OVS (months) (mean ± SD)	57.18 ± 41.32	54.11 ± 42.76	59.79 ± 35.86	76.00 ± 44.46	0.135
DFS (months) (mean ± SD)	33.32 ± 38.80	30.98 ± 40.15	26.71 ± 21.98	57.82 ± 51.31	0.031*

Statistical tests were selected based on the type of variable: the Kruskal-Wallis test was used for continuous variables such as DFS and OS, while one-way ANOVA was applied to other continuous variables (e.g., CA125 levels, age).

For categorical variables (e.g., FIGO stage, histopathology, menopausal status), Fisher's exact test was used. A significance level of p < 0.05 was applied throughout.

### Immunofluorescence

**Figure 1:**
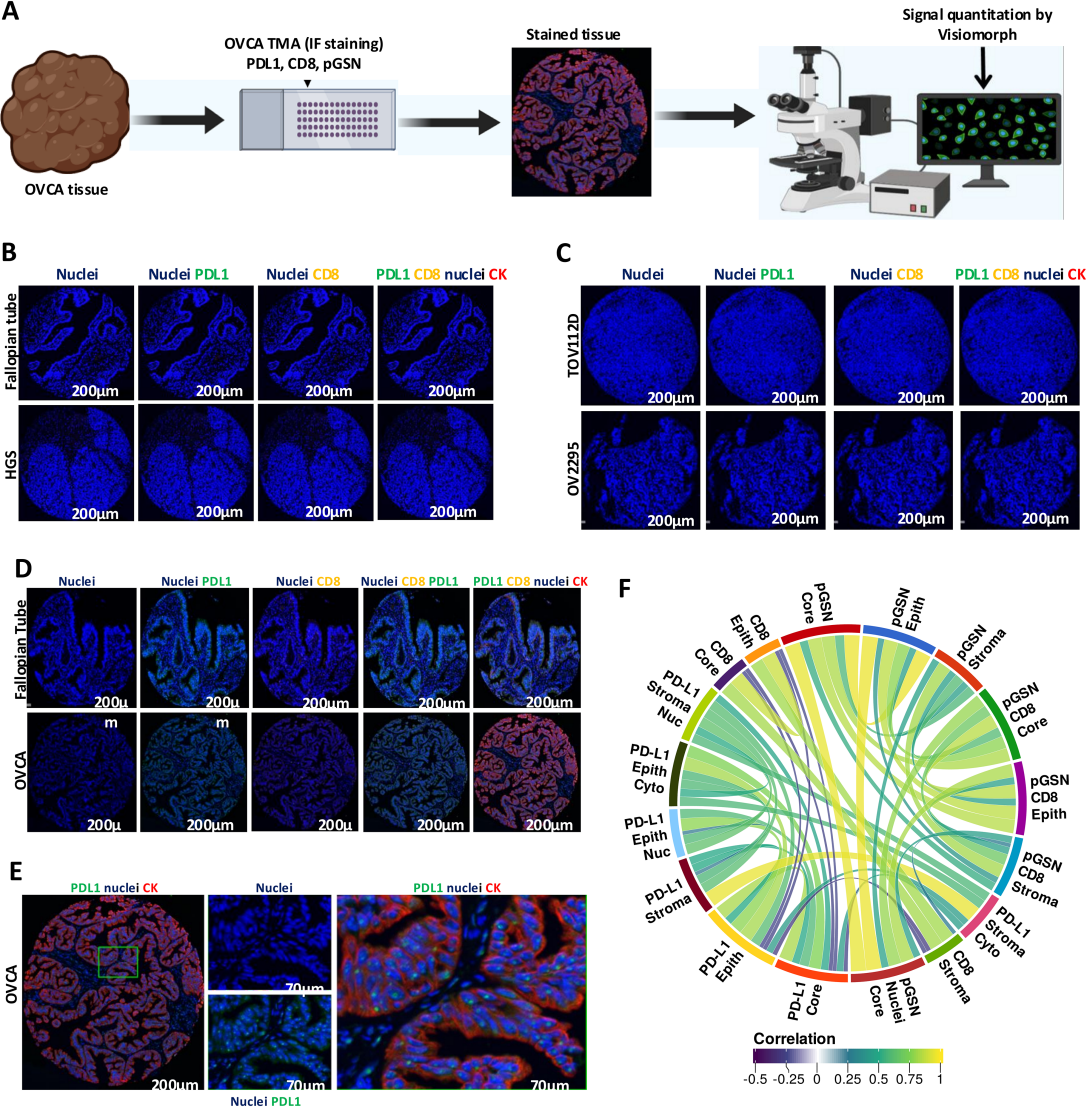
Localization of PDL1, pGSN and CD8+ T cells in ovarian cancer tissues. **(A)** Ovarian cancer tissues were immunostained with anti-cytokeratin(red), anti-PDL1 (green), and DAPI (blue) and MFI determined using Visiomorph. **(B)** Negative control validation of antibodies using normal fallopian tube and high grade serous (HGS) tissues. **(C)** Negative control validation of antibodies using cell line pellets. **(D)** immunostaining of normal fallopian tube and OVCA tissues with anti-cytokeratin(red), anti-PDL1 (green), CD8 (yellow) and DAPI (blue). **(E)** Localization of PDL1 in the nucleus of epithelial cells. **(F)** Correlative analysis of markers across tissue compartments and cellular locations. The circular visualization displays correlations between different markers (FDR < 0.01).

BenchMark XT automated stainer (Ventana Medical System Inc. Tucson, AZ) was used for the immunostaining of the OVCA Tissue Microarray (TMA). Tissues were taken through deparaffinization, antigen retrieval and antibody staining as previously described (9). Antibody cocktail against mouse anti-cytokeratin 7, 18 (Santa Cruz Biotechnology; ref. sc-5659) and 19 (NBP2-15186; ref. 1P170302; Novus Biologicals) coupled with Cy5 anti-mouse secondary antibody (Invitrogen; ref. A21236; lot #: 2119453) were used for the keratin staining. Anti-CD8 mouse monoclonal (Leica Biosystems; NCL-L-CD8-4B11) coupled with anti-mouse TRITC secondary antibody IgG2b 594 (Invitrogen; ref. A21145) were used for the CD8 staining. Anti-PDL1 rabbit antibody (Sigma-Aldrich; Ref. PRS4059) coupled with anti-rabbit AF488 secondary antibody (Invitrogen, ref. A11008; lot #: 2284594) were used for the PDL1 staining. In a separate panel, anti-pGSN mouse polyclonal (Antibodies online, ref. ABIN659182) coupled with anti-mouse AF647 (Invitrogen, ref. A21240; lot #: 2185066) were used for the pGSN staining. Dapi was used to stain the nuclei of the cells after which the slides were scanned with 20 x 0.7NA objective with a resolution of 0.3225um (VS110, Olympus, Center Valley, PA). The mean fluorescence intensity (MFI) of each marker were quantified in the region of interest (ROI) epithelium (keratin positive cells) and stroma (region negative for cytokeratin staining) identified by Visiomorph™ (Visiopharm, Denmark). The core region included both the epithelial and stroma compartments of the tumor tissue. The CD8+ T cells density score was determined by counting CD8 positive cells per 100.000 pixels of the ROI. To validate the specificity of the antibodies used in our study, we omitted the tissue marker-specific antibodies (**Figure 1**; **Supplementary Figure S1**).

### Quantitation of tissue markers

We utilized Visiomorph™ (Visiomorph, Denmark), a specialized software application designed for the analysis of digital pathology images and the identification of regions of interest (ROIs) within the tissue samples, specifically the epithelium (keratin-positive cells) and stroma (regions negative for cytokeratin staining). The mean fluorescence intensity (MFI) of each marker is then quantified within these ROIs. In the case of CD8+ T cells, we counted the number of CD8-positive cells within each tumor tissue ROI and established a CD8+ T cell density score. This score corresponds to the number of CD8-positive cells per 100,000 pixels of ROI, providing a standardized measure of immune cell infiltration. To ensure unbiased classification and measurement, all cores were batch-processed. This approach enables the simultaneous analysis of multiple samples, minimizing the potential for human error and ensuring consistency in the quantification of markers.

### Statistical analyses

Statistical analyses and data visualizations were performed using the R programming language (version 4.3.3). Associations between clinicopathologic parameters and tumor markers were analyzed using two-tailed Student’s t-tests, with statistical significance set at p-value < 0.05. To assess relationships between different tumor markers, we calculated Spearman’s rank correlation coefficients, a non-parametric measure of correlation, and visualized the results using the R package “corrplot” (version 0.95). Survival analyses were performed to evaluate the prognostic significance of the markers in relation to patient outcomes, including overall survival (OS) and disease-free survival (DFS). For robust survival modeling and visualization, these analyses utilized the R packages “survival” (version 3.7-0) and “survminer” (version 0.5). To dichotomize marker expression values, “survminer” package was used to calculate optimal cutoff points using maximally selected rank statistics. Kaplan-Meier (KM) survival curves were generated to compare survival distributions between groups, with statistical differences assessed using the log-rank test-based p-value. Hazard ratios (HR) for clinicopathological parameters and tissue marker expression levels were determined through univariate and multivariate Cox proportional hazard regression models. Results from these models were reported with corresponding 95% confidence intervals (CI). The multivariate survival analysis incorporated a range of clinicopathological parameters that are listed in **Table 2**.

**Table 2:** Cox proportional hazards model-based multivariable analyses of disease-free survival.

		HR		P value
Age		0.96 (*0.927 – 1.0*)	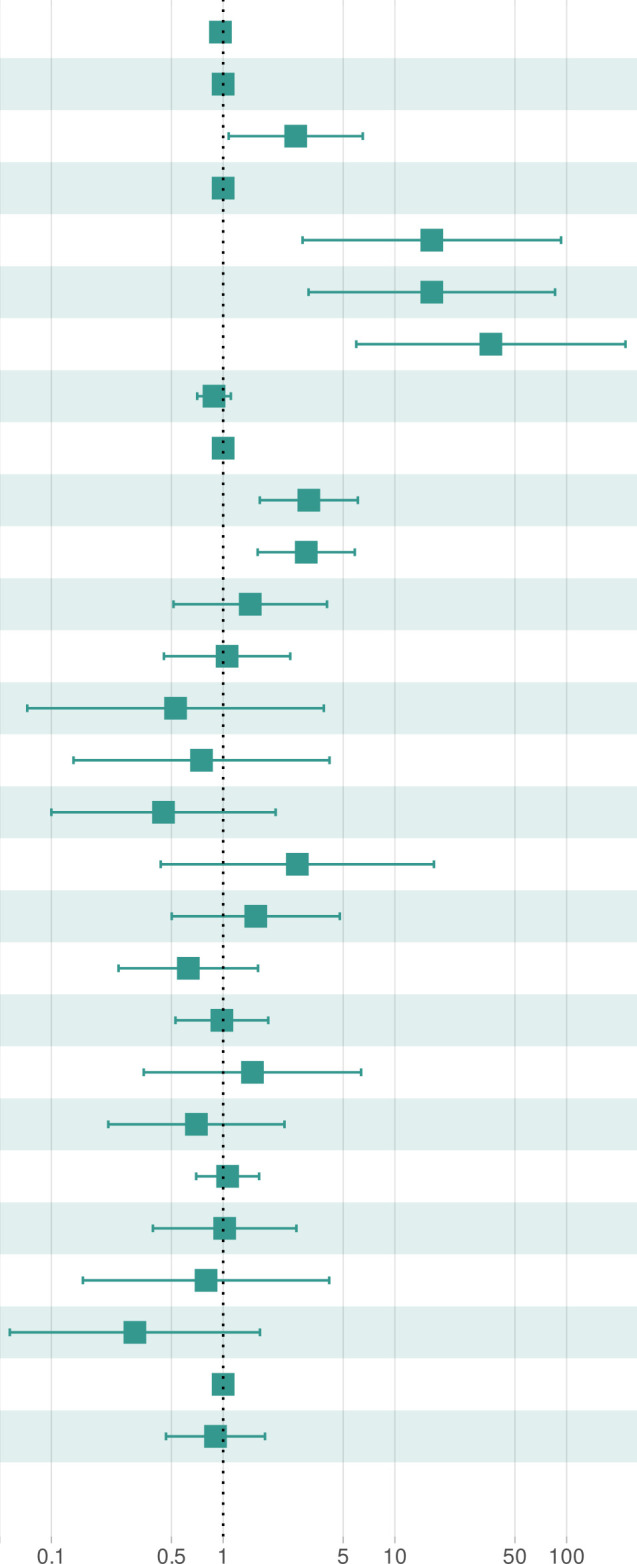	*0.074*
Menopause status	*0*	reference		
	*1*	2.65 (*1.077 – 6.5*)		0.034 *
FIGO	*1*	reference		
	*2*	16.40 (*2.900 – 92.8*)		0.002 **
	*3*	16.40 (*3.143 – 85.6*)		<0.001 ***
	*4*	36.20 (*5.956 – 220.0*)		<0.001 ***
CA125		0.88 (*0.706 – 1.1*)		*0.288*
RD Dichotomized	*0*	reference		
	*1*	3.15 (*1.634 – 6.1*)		<0.001 ***
Core PDL1		3.05 (*1.589 – 5.8*)		<0.001 ***
Epithelial PDL1		1.44 (*0.514 – 4.0*)		*0.489*
Stromal PDL1		1.05 (*0.452 – 2.5*)		*0.903*
Epithelial Nuclear PDL1		0.53 (*0.072 – 3.9*)		*0.529*
Epithelial Cytoplasmic PDL1		0.75 (*0.135 – 4.2*)		*0.741*
Stromal Nuclear PDL1		0.45 (*0.100 – 2.0*)		*0.297*
Stromal Cytoplasmic PDL1		2.70 (*0.433 – 16.9*)		*0.287*
Core CD8		1.55 (*0.502 – 4.8*)		*0.447*
Epithelial CD8		0.63 (*0.246 – 1.6*)		*0.327*
Stromal CD8		0.98 (*0.528 – 1.8*)		*0.957*
Core pGSN		1.48 (*0.345 – 6.4*)		*0.597*
Epithelial pGSN		0.70 (*0.214 – 2.3*)		*0.552*
Stromal pGSN		1.06 (*0.696 – 1.6*)		*0.78*
Core Nuclear pGSN		1.02 (*0.390 – 2.7*)		*0.967*
Epithelial:Cyotplasmic Ratio PDL1		0.80 (*0.153 – 4.1*)		*0.786*
Stromal:Cytoplasmic Ratio PDL1		0.31 (*0.057 – 1.6*)		*0.167*
BRCA1/2 Status	Wild-type	reference		
	Mutant	0.90 (*0.465 – 1.8*)		*0.762*

### Machine learning based predictive modeling of chemoresistance and patient survival

We conducted a machine learning analysis to evaluate the utility of tissue marker expression levels in predicting chemoresistance. Chemoresistance was defined as a progression-free interval (PFI) of ≤ 12 months, while chemosensitivity was defined as a PFI of > 12 months. Additionally, we assessed treatment responsiveness using a 6-month threshold. To build a machine learning model for chemoresistance prediction, we applied the Elastic Net algorithm, utilizing tissue marker expression values alongside clinicopathological parameters as features. We implemented a four-fold cross-validation strategy to assess model performance. In each cross-validation round, the data were spitted into a training set (75% of the data) and a validation set (25% of the data). To address class imbalance during model training, oversampling of the minority class was performed to create a more balanced representation of both classes in the dataset. The Elastic Net parameters, alpha and lambda, were optimized using a grid search approach on the training dataset. To comprehensively assess model performance, multiple evaluation metrics were employed, including sensitivity, specificity, F1-score, and area under the curve (AUC). After optimization, we predicted outcomes for the validation data. This process was repeated four times. Posterior class probabilities from each validation set were concatenated, and the results were visualized using receiver operating characteristic (ROC) plots. Area under the curve (AUC) values were computed to quantify model performance. This analysis was performed using the R package “caret” (version 6.0-94). We trained and validated a separate machine learning model for predicting disease-free survival (DFS). For this purpose, we used a regularized Cox regression algorithm implemented in the R package “glmnet” (version 4.1-8). Cross-validation strategy was applied as described earlier for model training and testing. Results were visualized using the R package “survivalROC” (version 1.0.3.1).

## Results

### Patients’ characteristics

Board certified gynecologic pathologists performed the tumor staging and pathology for the 208 OVCA tissues used in the study. Details of the patients’ characteristics according to their *BRCA* status are described in **Table 1**. The median age for *BRCA* non-carriers, *BRCA1* carriers and *BRCA2* carriers are 63.14, 51.43 and 58 years, respectively. Patients enrolled in the study received no radiotherapy nor neoadjuvant chemotherapy prior to sample collection at surgery with eighty-six (86) of the patients achieving optimal cytoreduction. Optimal cytoreduction was defined as residual tumor nodules measuring ≤1 cm in maximum diameter, whereas suboptimal cytoreduction was defined as the presence of residual tumor nodules >1 cm following surgery.

### Tissue localization of PD-L1, pGSN and CD8+ T cells in normal fallopian tube, OVCA tissues and cell lines

Anti-PD-L1 immunotherapy has shown modest response in OVCA patients despite significant treatment responses in other solid cancers (15, 20–22, 29). The reason behind this is not fully understood. Here, we investigated how PD-L1 correlates with pGSN, a pro-survival protein, to suppress the immune system. PD-L1, pGSN and infiltrated CD8+ T cells were immunostained on a tissue microarray constructed with 208 OVCA tissues (**Table 1** and **Figure 1A**). The staining specificity of the tissue marker antibodies are shown in **Supplementary Figures 1A–H**). The staining of the tissue markers was optimized using normal fallopian tube, OVCA tissues and OVCA cell line-derived pellets (**Figures 1B–E**). The mean fluorescence intensity (MFI) was determined using the digital image analysis tool, Visiomorph. We observed no false staining or auto fluorescence in the normal and malignant tissues when no tissue marker antibody was used (**Figures 1B, C**). Although pGSN expression was scarcely observed in the epithelial and stromal compartments of the normal fallopian tube tissue, CD8+ T cells and PD-L1 were minimally detected (**Figure 1D**). Meanwhile, pGSN and PD-L1 were significantly detectable in OVCA tissues with PD-L1 localization observed in the nuclei of the epithelial compartment (**Figures 1D, E**). For each tissue marker expression, there was a positive correlation between compartments whereas a negative correlation was observed between PD-L1 and CD8+ T cells regardless of the tissue compartment and cellular location (**Figure 1F**; **Supplementary Figure S1**).

### Increased nuclear PD-L1 and pGSN expression in the epithelial and stromal compartments is associated with reduced survival impact of infiltrated CD8+ T cells

**Figure 2:**
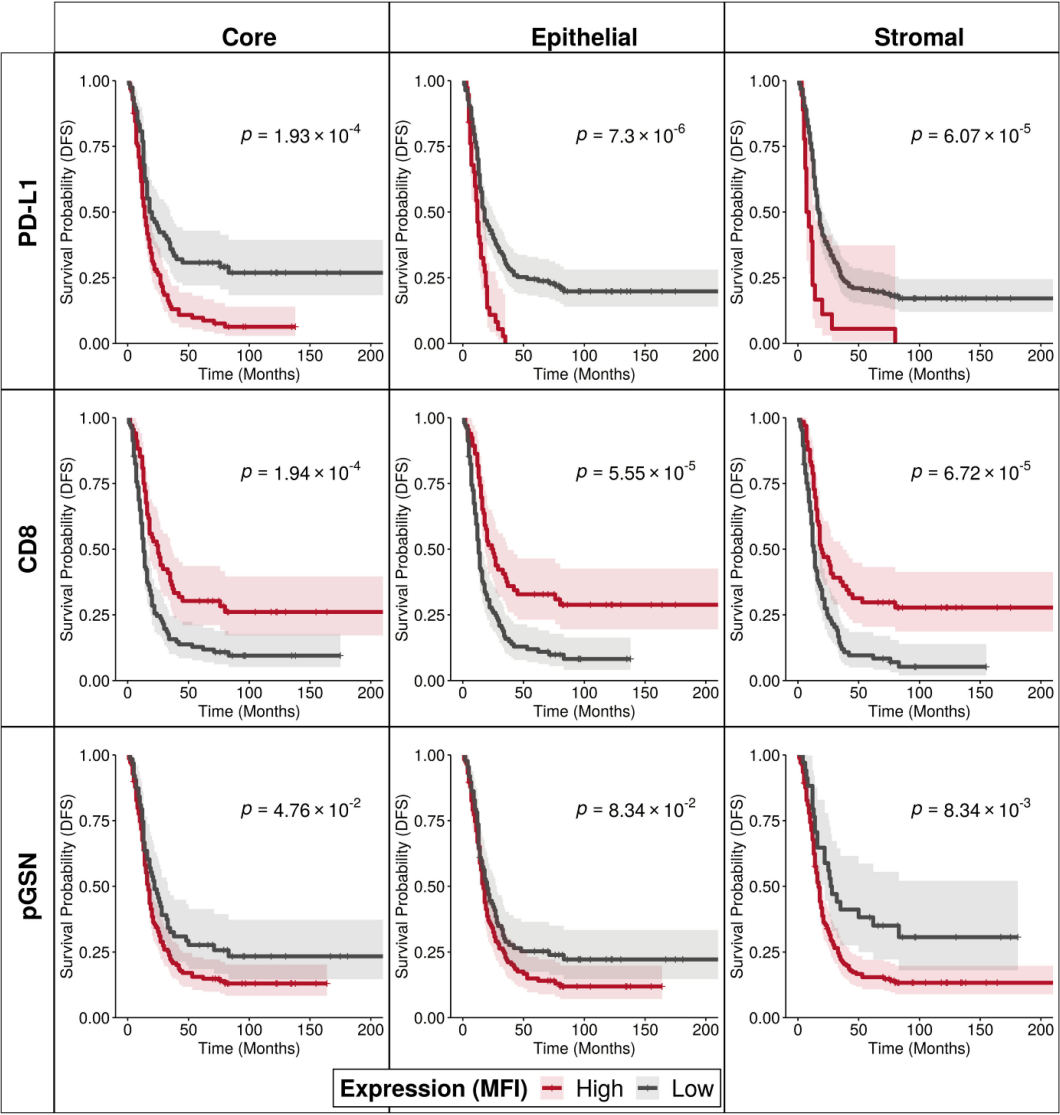
Increased nuclear PD-L1 and pGSN expressions are associated with poor patient outcome. Kaplan–Meier survival curves depicting disease-free survival for high and low expression levels of PD-L1, CD8, and pGSN proteins (indicated by rows) within the core, epithelial, and stromal compartments (indicated by columns). Expression values for each protein were dichotomized, and survival distributions between groups were compared using the log-rank test.

To determine the prognostic impact of nuclear PD-L1, pGSN and CD8+ T cells, MFI of their localization was detected in both the epithelial and stroma compartments. Using an MFI cut-off for each tissue marker, patients were dichotomized into high and low groups. Kaplan-Meier survival curves and log-rank tests were used to compare the survival distributions between the groups. Our results show that OVCA patients with increased nuclear PD-L1 regardless of the tissue compartment had shortened DFS and OS compared with those with decreased nuclear PD-L1 (**Figure 2**; **Supplementary Figures S2, S3**). Interestingly, we observed a significant association between increased nuclear PD-L1 and poor patient survival within the epithelial (p-value=7.3 x 10^-6^) and stromal (p-value=6.07 x 10^-5)^ compartments (**Figure 2**; **Supplementary Figures S2, S3**). While increased infiltration of CD8+ T cells were associated with improved patient survival, patients with increased pGSN expression had shortened survival compared with others with decreased pGSN expression (**Figure 2**; **Supplementary Figure S4–S6**).

**Figure 3:**
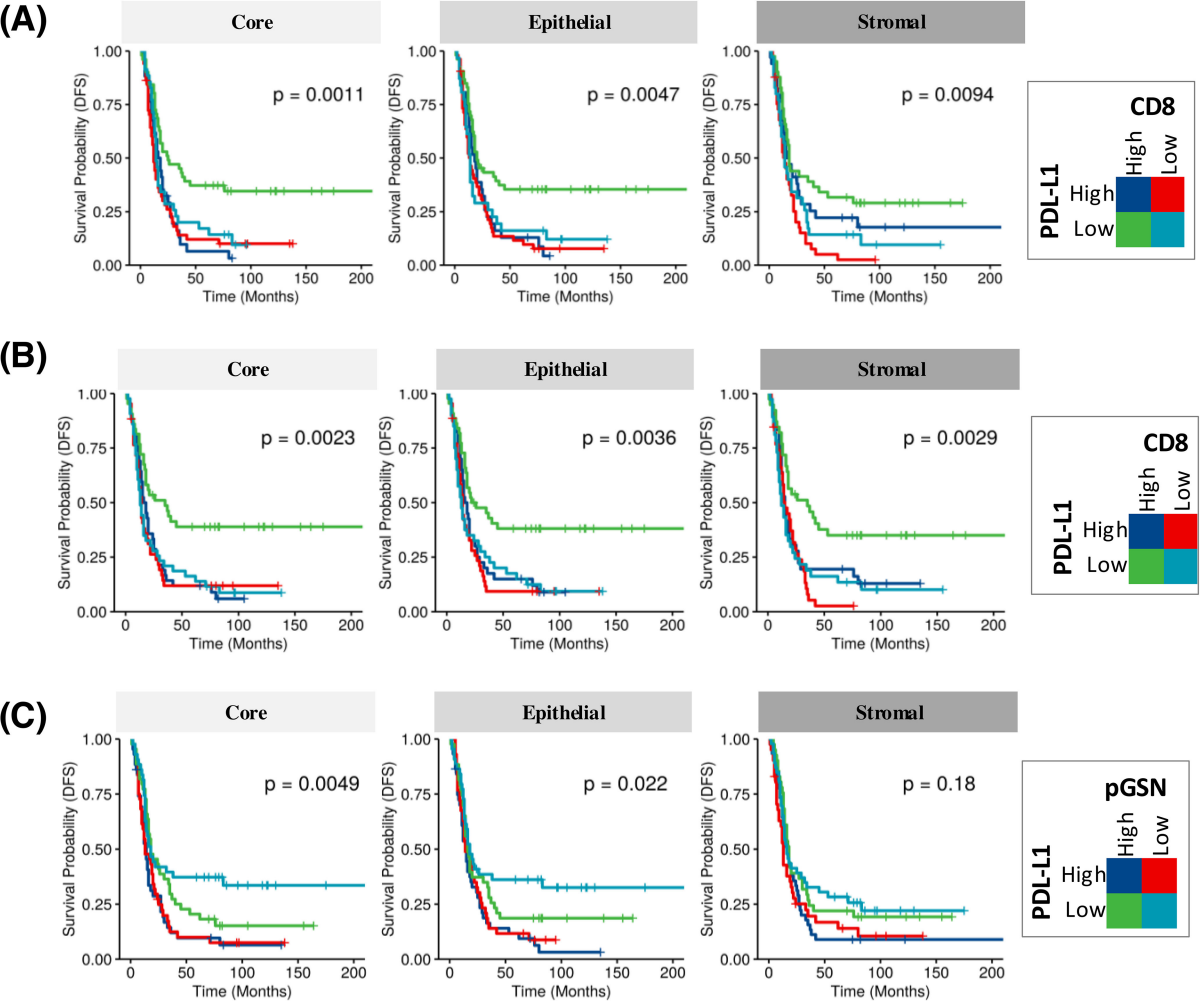
Increased nuclear PD-L1 and pGSN hinder the anti-tumor benefits of infiltrated CD8+ T cells in the OVCA microenvironment and associated with disease-free survival (DFS). KM plot in **(A)** shows PD-L1 expression in combination with infiltrated CD8+ T cells in the core, epithelial and stromal compartments. **(B)** Epithelial nuclear PD-L1 expression in combination with CD8+ T cell presence in the core, epithelium, and stroma. **(C)** PD-L1 expression in combination with pGSN expression in the core, epithelium, and stroma. For the analysis, samples were classified into four groups based on the expression levels of two markers. Each marker was dichotomized into high and low expression, resulting in four groups: high-high, high-low, low-high, and low-low. Kaplan–Meier survival plots depict disease-free survival probabilities across these groups for the respective markers. Survival distributions were compared using the log-rank test.

We further determined if the interaction of these markers in the core, epithelial and stromal compartments affect the survival of patients. Two markers were combined at a time and patients were categorized into four (4) groups based on low and high expressions of the markers (**Figure 3**; **Supplementary Figures S7–S9**). When nuclear PD-L1 and CD8 were combined in the analyses, patients with low nuclear PD-L1 and high CD8 had the most survival benefits (**Figure 3A**, log-rank test p-value=0.001, 0.004 and 0.009 for core, epithelial and stromal, respectively). Similar results were observed within the epithelial compartment (**Figure 3B**, log-rank test p-value=0.002, 0.003 and 0.002 for core, epithelial and stromal, respectively). The survival benefit of CD8+ T cells was however, suppressed when nuclear PD-L1 is increased, suggesting that elevated nuclear PD-L1 may suppress the survival impact of infiltrated CD8+ T cells (**Figures 3A, B**). A similar observation was made with overall survival when the analyses were performed using nuclear PD-L1 in the epithelial and stromal compartments (**Supplementary Figure S9**). When analyzing nuclear PD-L1 and pGSN together, patients with low nuclear PD-L1 and low pGSN showed the highest survival improvement, while those with high nuclear PD-L1 and high pGSN had the poorest survival outcomes (**Figure 3C** log-rank test p-value=0.004, 0.02 and 0.18 for core, epithelial and stromal, respectively). This effect was significant in the epithelial compartment but not the stroma (**Supplementary Figure S7**). A similar trend was observed when the analyses was stratified by nuclear PD-L1 in the epithelial and stromal compartments (**Supplementary Figures S8, S9**), suggesting that co-expression of pGSN and nuclear PD-L1 is associated with poor prognosis. We have previously demonstrated that pGSN uptake by T cells induces apoptosis, contributing to immuno-suppression and chemoresistance. We confirmed this phenomenon as we observed increased uptake of pGSN in CD8+ T cells in patients with poor survival (**Supplementary Figure S10**).

### *BRCA1* mutations are associated with increased nuclear PD-L1 expression

**Figure 4:**
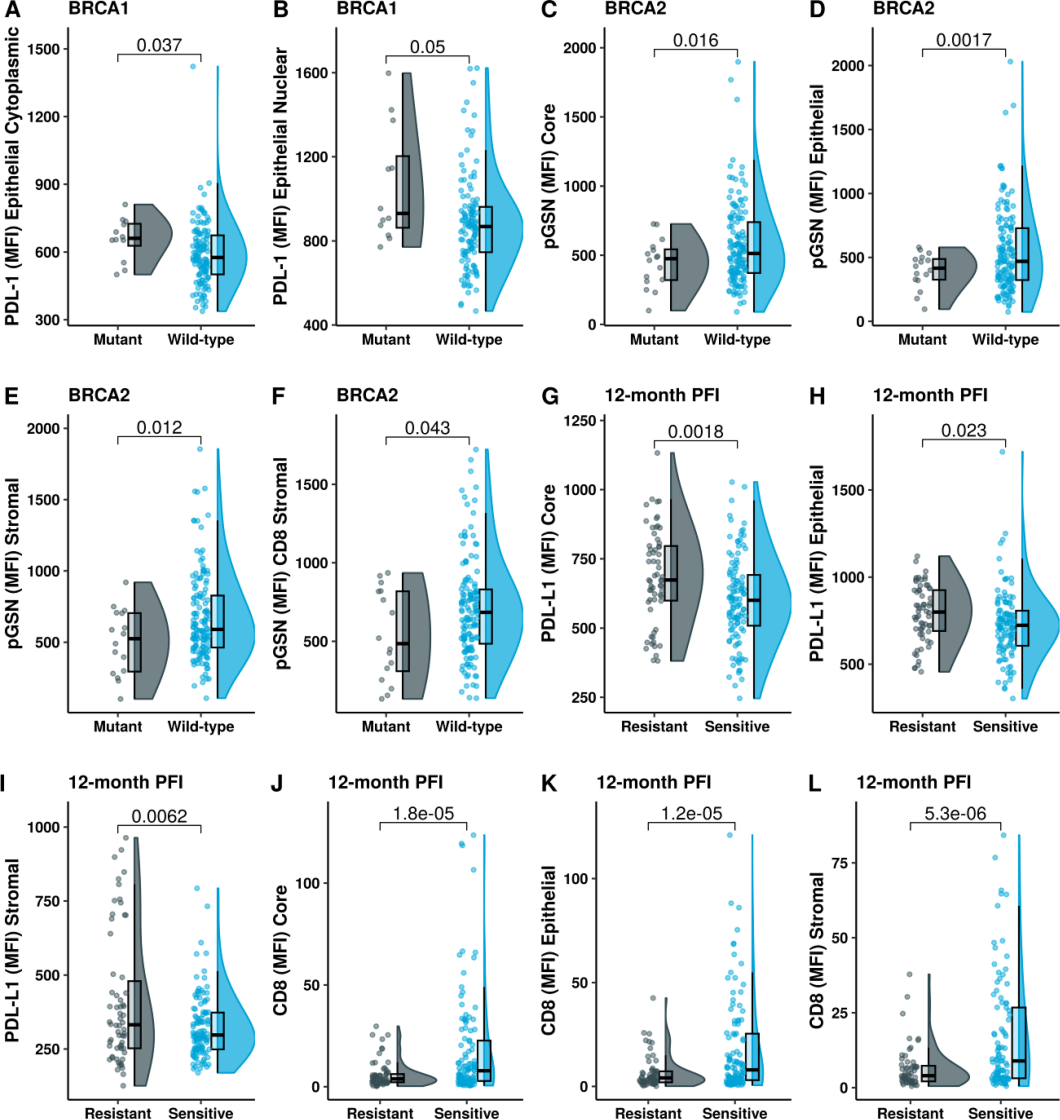
BRCA1 mutation is associated with increased levels of PD-L1 and chemoresistance. Raincloud plots show **(A)** epithelial cytoplasm PD-L1 and **(B)** epithelial nucleus PD-L1 levels, stratified by BRCA1 mutation status. Raincloud plots illustrate **(C)** pGSN core, **(D)** pGSN epithelial, **(E)** pGSN stromal and **(F)** pGSN in CD8+ T-cell stromal regions, with protein expression stratified by BRCA2 mutation status. Expression level of **(G)** PD-L1 core, **(H)** PD-L1 epithelial, **(I)** PD-L1 stromal, **(J)** CD8 core, **(K)** CD8 epithelial and **(L)** CD8 stromal in chemosensitivity (PFI > 12 months) and chemoresistance (PFI ≤ 12 months). The median difference between the two groups was compared using Student’s t-test and p-value is indicated on the top.

Given the clinical importance of *BRCA* status in determining the risk for OVCA, we stratified patients based on their *BRCA* status and compared their tissue PD-L1, pGSN and CD8+ T cell levels. Our results show that the carriers of *BRCA1* mutation had increased levels of epithelial cytoplasmic and epithelial nuclear PD-L1 compared with non-carriers (**Figures 4A, B**). All other comparisons were not significant (**Supplementary Figures S11–S13**). We also found that the *BRCA2* mutation carriers had decreased levels of epithelial and stromal pGSN compared with non-careers (**Figures 4C–F**). All other *BRCA2* comparisons were not significant (**Supplementary Figures S14–S16**). These findings reveal *BRCA* mutation-specific association with both PD-L1 and pGSN expressions.

### Increased PD-L1 nuclear localization is associated with chemoresistance

We next assessed the association between PD-L1 nuclear localization and responsiveness to chemotherapy in patient. Chemoresistance was defined as progression free interval (PFI) ≤ 12 months and chemosensitivity as PFI > 12 months. We also stratified treatment responsiveness using 6 months as the criteria. Chemoresistant patients (PFI ≤12 months) had increased levels of PD-L1 compared with chemosensitive patients (PFI > 12 months) in both the epithelial and stromal compartments of the tissue (**Figures 4G–I**; **Supplementary Figures S17–S21**). There was no significant difference observed with nuclear and cytoplasmic PD-L1 when the 6 months criteria were used (**Supplementary Figures S19–S21**). On the contrary, chemosensitive patients had increased infiltration of CD8+ T cells in the epithelial and stromal compartments (**Figures 4J–L**). This difference was regardless of the criteria used for defining treatment response (6- and 12-months, **Supplementary Figure S20**). When using 6-months PFI criteria, we observed significant association with chemosensitivity for stromal pGSN, and pGSN colocalized in CD8 T cells in the epithelial and stromal compartments (**Supplementary Figure S21**).

**Figure 5:**
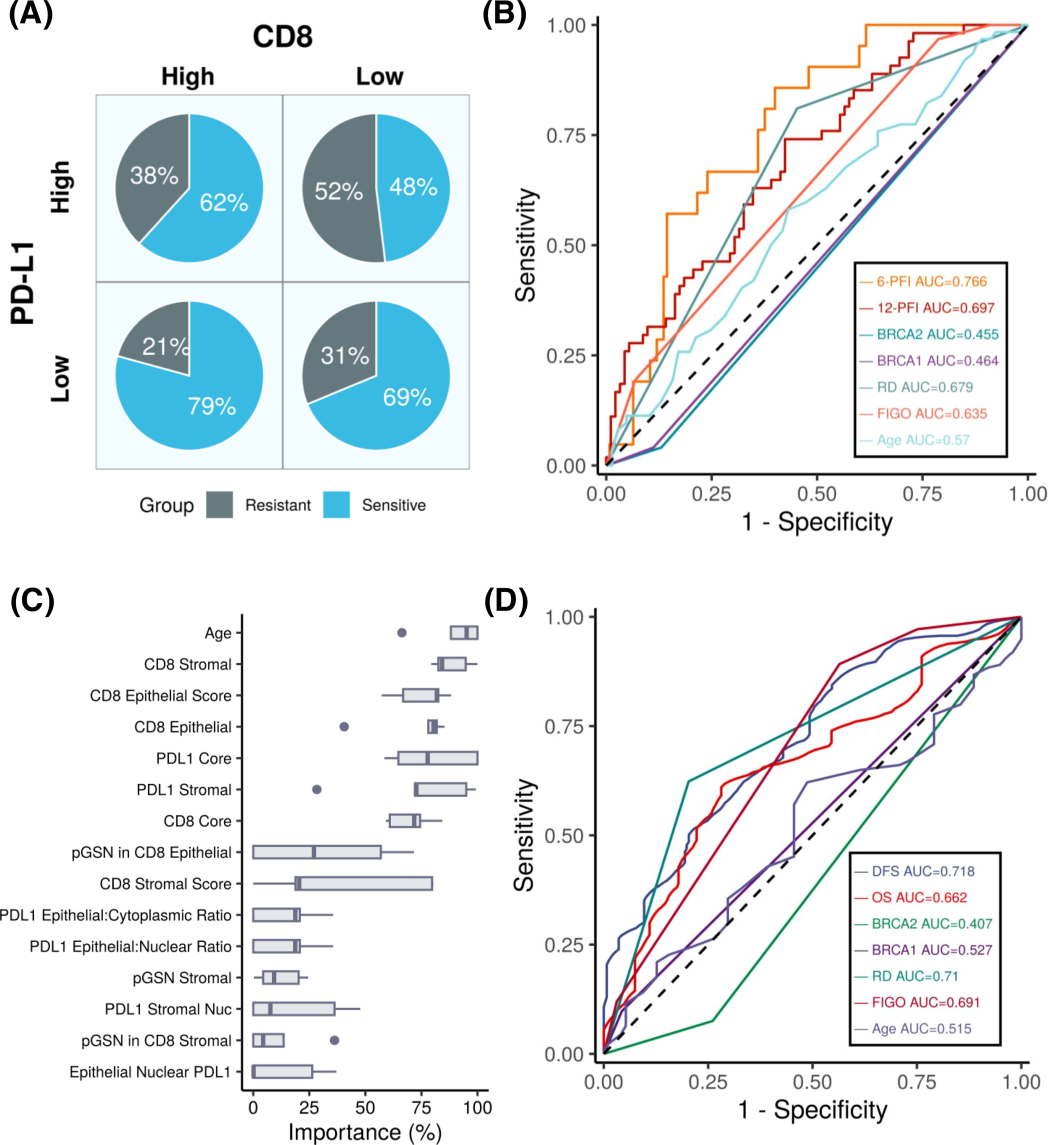
PD-L1 expression predicts patient outcomes in ovarian cancer. **(A)** Pie chart depicting the percentages of chemosensitive and chemoresistant patients within PD-L1 high/low and CD8 high/low groups. **(B)** ROC plot showing the performance of our machine learning models compared to other variables in predicting progression-free interval (PFI) at 6 months and 12 months. Results indicating that the PD-L1 based model achieves the highest predictive accuracy, with AUC values of 0.76 and 0.69 for 6- month and 12-month PFI, respectively. **(C)** Boxplots showing variable importance in the optimized machine learning model, highlighting PD-L1 core and PD-L1 stroma as among the most important variables. **(D)** Survival ROC plot illustrating that our machine learning model achieves an AUC of 0.71 and outperforms other variables when predicting DFS.

We next examined the combined effect of PD-L1 and CD8+ T cells on chemoresistance (**Figure 5A**). In the high PD-L1 and low CD8 group, 52% of patients were chemoresistant, whereas only 21% of patients in the low PD-L1 and high CD8 group were chemoresistant. These findings highlight the poor prognostic characteristic of PD-L1 expression in OVCA patients as well as suggest an inverse relationship between PD-L1 expression and CD8+ T cell levels in predicting chemoresistance.

### Prognostic impact of PD-L1, pGSN, CD8+ T cells

To determine the prognostic impact of the tissue markers together with clinicopathological parameters in predicting DFS and OS, we used univariate and multivariate Cox regression analyses (**Table 2**; **Supplementary Tables S1**–**S3**). From the univariate Cox regression analysis, we observed that age, FIGO stages 3 and 4, residual disease, PD-L1 (core, epithelial and stroma), nuclear PD-L1 (epithelial and stroma), cytoplasm PD-L1 (epithelial and stroma), CD8 (core, epithelial and stroma), pGSN core, epithelial pGSN and nuclear pGSN were significantly associated with OS (**Supplementary Table S1**). For DFS, FIGO stages 3 and 4, residual disease, PD-L1 (core, epithelial and stroma), nuclear PD-L1 (epithelial and stroma), cytoplasm PD-L1 (epithelial and stroma), CD8 (core, epithelial and stroma) and *BRCA2* status significantly predicted DFS (**Supplementary Table S2**). In the multivariate Cox regression analysis, menopausal status, FIGO stage, residual disease, and PD-L1 core were significant predictors of disease-free survival (**Table 2**). These findings were consistent when the analysis performed for overall survival (**Supplementary Table S3**). Next, we evaluated the chemosensitivity prediction and prognostic potential of the selected markers. Using marker expression values and clinical data, we developed an Elastic Net-based machine learning model to predict chemoresistance (PFI ≤ 12 months) and chemosensitivity (PFI > 12 months). Cross-validation demonstrated that the model achieved an area under the curve (AUC) value of 0.697 (**Figure 5B**). Notably, when predicting PFI at 6 months, the model’s performance improved, yielding an AUC value of 0.766. This model outperformed other clinical variables in predicting chemoresistance. To identify the most influential variables in the machine learning model, we conducted a variable importance analysis (**Figure 5C**). The results indicated that PD-L1 core, PD-L1 stroma and different CD8 markers were among the most critical features selected by the model. Additionally, we trained a separate Elastic Net-based model to predict disease-free survival (DFS) using protein marker expression and clinical data (**Figure 5D**). Cross-validation showed that the model achieved an AUC of 0.718 for DFS prediction and 0.662 for OS prediction. Comparisons with AUC values derived from clinical variables alone confirmed that DFS prediction demonstrated superior performance. In summary, our findings highlight that the markers such as PD-L1, pGSN, and CD8+ T cells are robust predictors of chemoresistance and can reliably predict patient survival outcomes.

## Discussion

Anti-PD-L1 immunotherapy has achieved only a modest therapeutic success in OVCA patients despite significant therapeutic success in other solid tumors (21, 22). Although there are extensive studies on cyto-membranal PD-L1, scarce attention has been given to the nuclear localization of PD-L1 and its clinical significance in OVCA and other cancer type (26, 28). In this study, our findings highlight the prognostic value of nuclear PD-L1 and its relationship with pGSN and CD8+ T cells. Furthermore, our study provides a potential explanation for the modest therapeutic response observed with anti-PD-L1 therapies.

Increased PD-L1 expression in the epithelial and stromal compartments of the tissues was associated with shortened OS and DFS. In this context our findings are consistent with previous studies that have reported similar findings in other cancers (18, 30, 31). When stratified by cellular location, we observed the most significant effect with nuclear PD-L1, suggesting a potential prognostic value. Although increased infiltration of CD8+ T cells provided survival benefits to patients, these survival benefits were significantly hindered by nuclear PD-L1 elevation, a phenomenon that we had previously seen with pGSN also (9). Interestingly, patients with elevated pGSN and nuclear PD-L1 had the worst survival, suggesting a synergistic pro-tumor effect of both markers. Chemoresistance presents as a major obstacle in achieving therapeutic success in OVCA patients. Our previous studies have shown a significant association between elevated pGSN and chemoresistance (8, 10, 12). In addition to confirming this phenomenon in our current study, we have also demonstrated a significant association between epithelial expression of PD-L1 and chemoresistance.

Anti-PD-L1 antibodies bind and inhibit PD-L1 on the surface of cancer cells, thus, preventing its interaction with PD-1 (32). The authors observed that the inhibition was associated with increased presence of nuclear PD-L1, thus, rendering the therapeutic effects of anti-PD-L1 ineffective. Nuclear PD-L1 has been detected as a poor prognostic marker in breast cancer, lung adenocarcinoma, renal cell carcinoma, hepatocellular carcinoma, and prostate cancer (24–28). In uveal melanoma, nuclear PD-L1 promotes early growth response-1 (EGR1)-mediated angiogenesis and tumorigenesis (28). Yang Gao, et al., have shown that PD-L1 translocate into the nucleus via an acetylation-dependent pathway and blocking its translocation resulted in an enhanced therapeutic efficacy of PD-1/PD-L1 inhibitor treatment (24). pGSN induces HIF1alpha-mediated chemoresistance in OVCA cells while HIF1alpha drives PD-L1 transcription leading to poor anti-PD-L1 response (8, 26, 33). We therefore hypothesize that pGSN activates HIF1alpha-mediated PD-L1 transcription resulting in the expression of tumor promoting and immune-suppressive genes, a process leading to tumor recurrence, chemoresistance and poor overall survival.

We also compared the relative levels of PD-L1, pGSN and CD8+ T cells between carriers and non-carriers of *BRCA1/2* mutations as well as how these mutations relate to chemoresistance and patient survival. We found that PD-L1 was elevated in *BRCA1* mutation carriers while pGSN was downregulated in *BRCA2* mutation carriers. *BRCA1/2* characterization may therefore provide clinicians with prior information about pGSN and PD-L1 expression in the OVCA microenvironment in personalized therapeutic options.

The current study despite its key findings, has a few limitations. The tissues were retrospectively collected with no prospective collections. The Canadian Ovarian Experimental Unified Resource (COEUR) manages the collection of tissues and adheres to the standards defined by the Canadian Tissue Repository Network (CTRNet), which ensures the quality of the biological material (34). Thus, we don’t anticipate any interferences with our staining. Although we attempted to mitigate biases associated with treatment heterogeneity by controlling for critical factors, such as genetic mutations and relevant clinical parameters in statistical analysis, treatment variability may still have influenced survival outcomes- a common limitation in cohort-based studies. However, our methodology, analyzing both PFS and OS, along with rigorous adjustment for treatment-related factors has helped to substantially reduce the potential impact of these biases. We did not perform functional experiments, which restricts our ability to confirm the mechanistic relevance of the observed associations. Lastly, pGSN staining was conducted on a separate tissue panel, preventing us from providing a co-localization image with PD-L1 or CD8+ T cells within the same tumor microenvironment. Future studies will investigate the *in vitro* and *in vivo* mechanistic interaction between nuclear PD-L1 and pGSN and how that promotes chemoresistance in OVCA.

For the first time, we have provided convincing evidence about the detection and prognostic value of nuclear PD-L1 in OVCA. We have shown that elevated PD-L1 together with pGSN suppress the anti-tumor functions of CD8+ T cell contributing to OVCA recurrence, chemoresistance and poor overall survival.

## Data Availability

The original contributions presented in the study are included in the article/**Supplementary Material**. Further inquiries can be directed to the corresponding authors.
