# Supplementary material for: Spotlight on nuclear PD-L1 in ovarian cancer chemoresistance: hidden but mighty
Source: Front Immunol. 2025 Jul 14;16:1543529. doi: 10.3389/fimmu.2025.1543529 (PMC12301380; doi:10.3389/fimmu.2025.1543529)
Supplement: Supplementary file 1 [file DataSheet1.pdf]

Spotlight on nuclear PD-L1 in ovarian cancer chemoresistance: “hidden but mighty”

Supplementary Figures and Tables

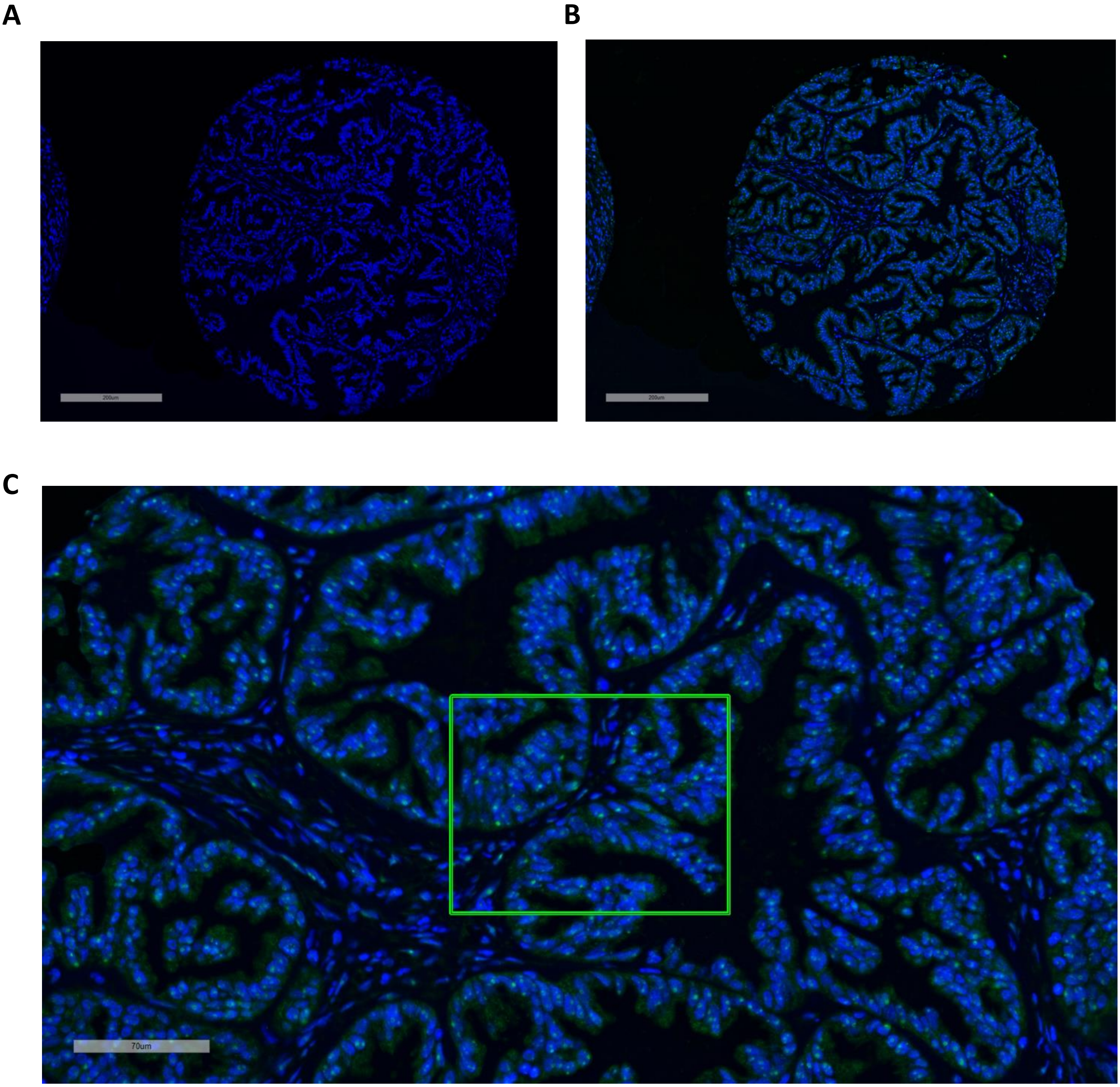
A
B
200um
200um
C
70um

D

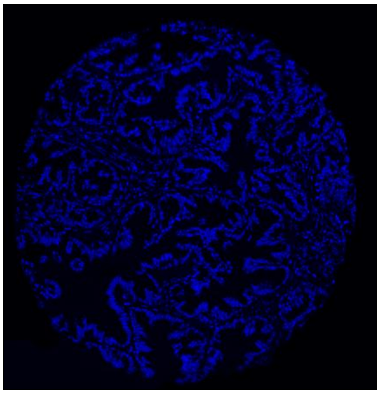

E

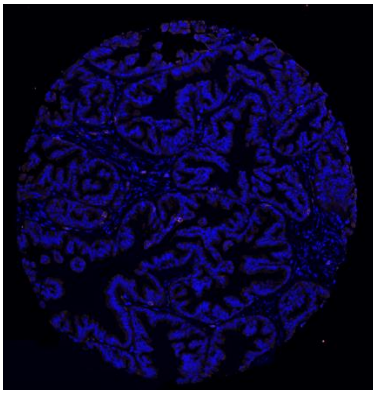

F

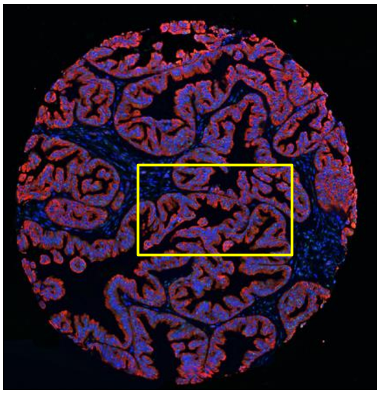

G

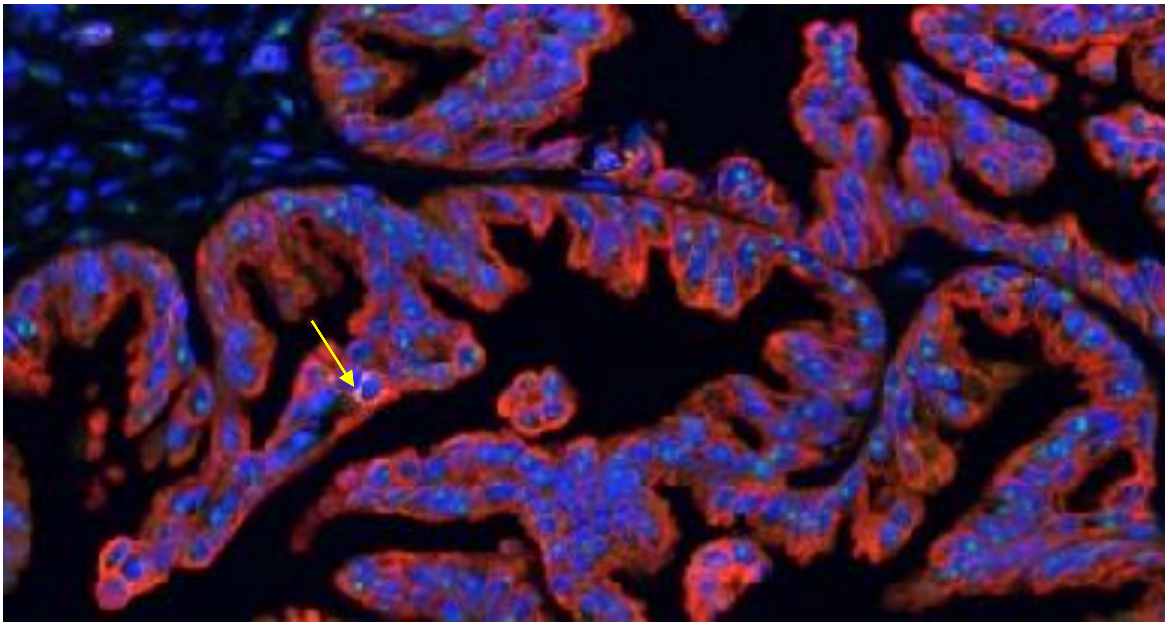

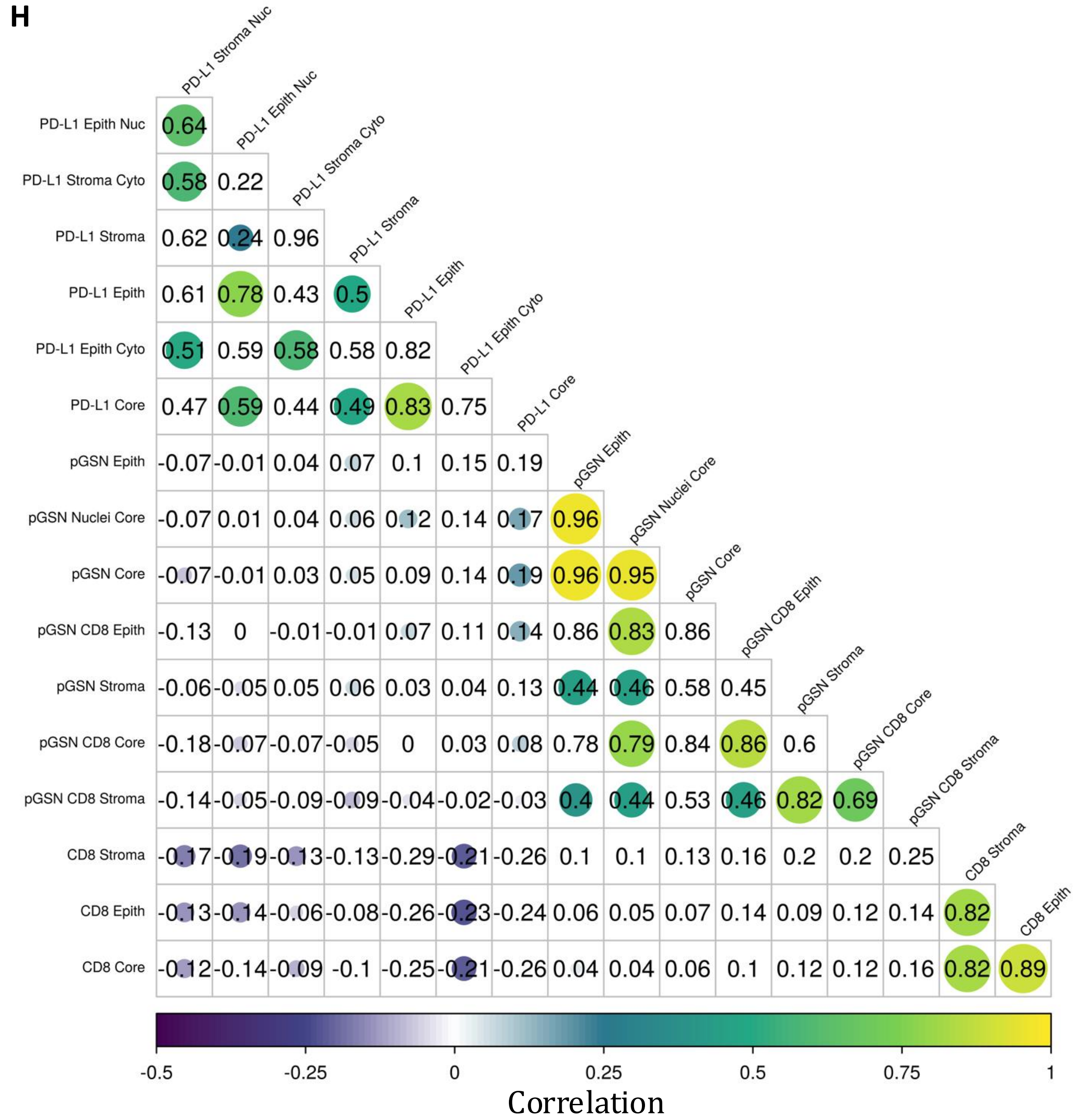

Supplementary Figure S1: Nuclear PD-L1 and CD8 T cell staining and correlative analysis. (A) Negative stain with no PD-L1 specific antibody. Nuclei is stained with dapi (blue). (B) PD-L1 specific staining. (C) PD-L1 specific staining (yellow box is the area shown in Figure 1). (D) Negative stain with no PD-L1 and CD8 specific antibodies. Nuclei is stained with dapi (blue). (E) CD8 T cells specific staining. (F) PD-L1, CD8 T cells and cytokeratin specific staining. (G) Zoomed image of PD-L1 and CD8 co-localization (yellow arrow). (H) Correlative analysis of markers across tissue compartments and cellular locations. Spearman's rank correlation coefficient was computed between each markers. Colors indicate the correlation coefficient that has statical significance (p-value<0.05).

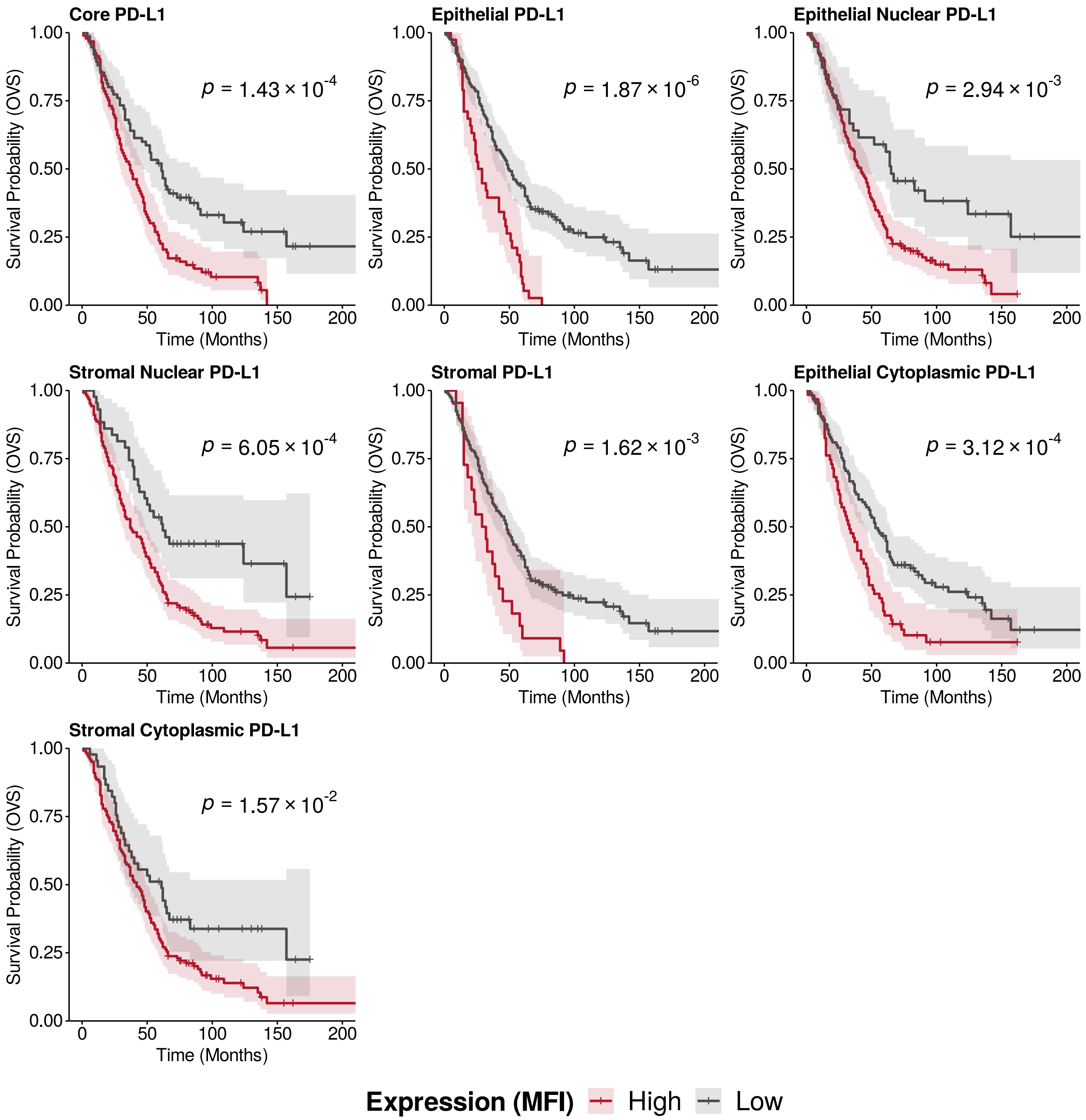

Supplementary Fig. S2: PD-L1 expression and overall patient survival (OS). Kaplan-Meier survival plots display survival outcomes for dichotomized PD-L1 expression groups (low vs. high). P-values were computed using the log-rank test.

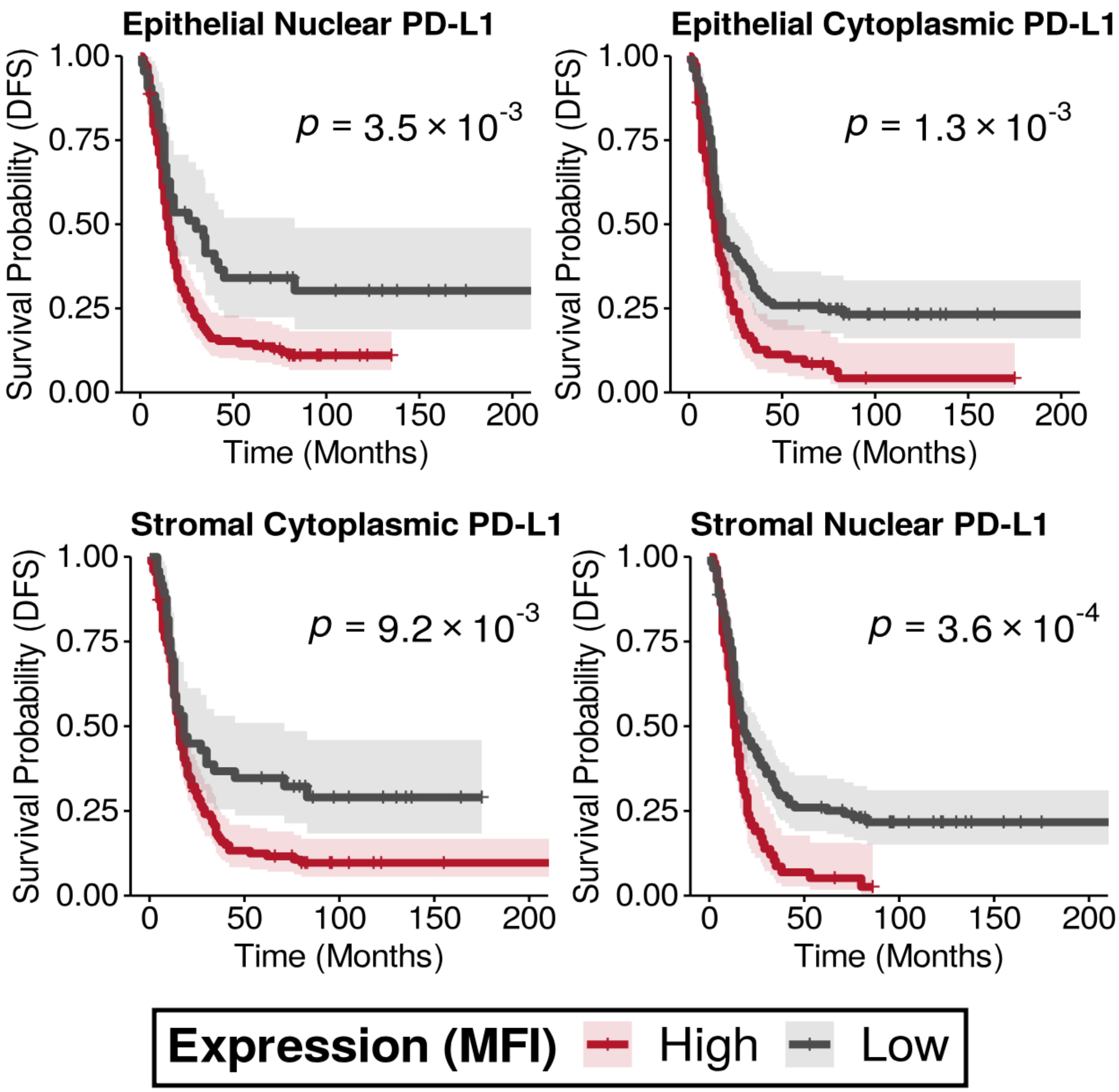

Supplementary Fig. S3: Epithelial and stromal PD-L1 expression and disease-free survival (DFS) in different cellular compartments. Kaplan-Meier survival plots display survival outcomes for dichotomized PD-L1 expression groups (low vs. high). P-values were computed using the log-rank test.

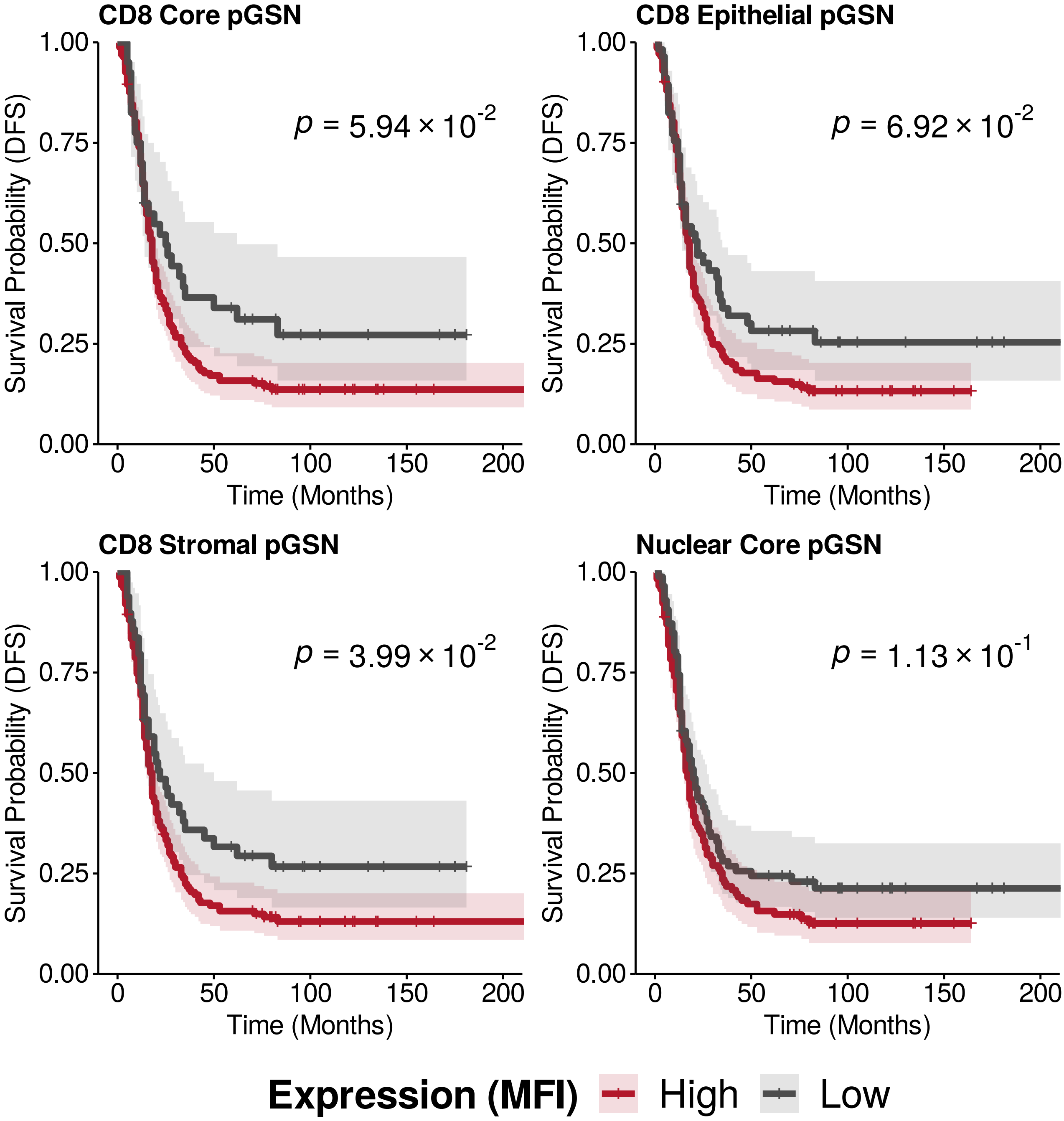

Supplementary Fig. S4: pGSN localization in T cells and disease-free survival (DFS). Kaplan-Meier survival plots display survival outcomes for dichotomized pGSN-CD8 co-localization groups (low vs. high). P-values were computed using the log-rank test.

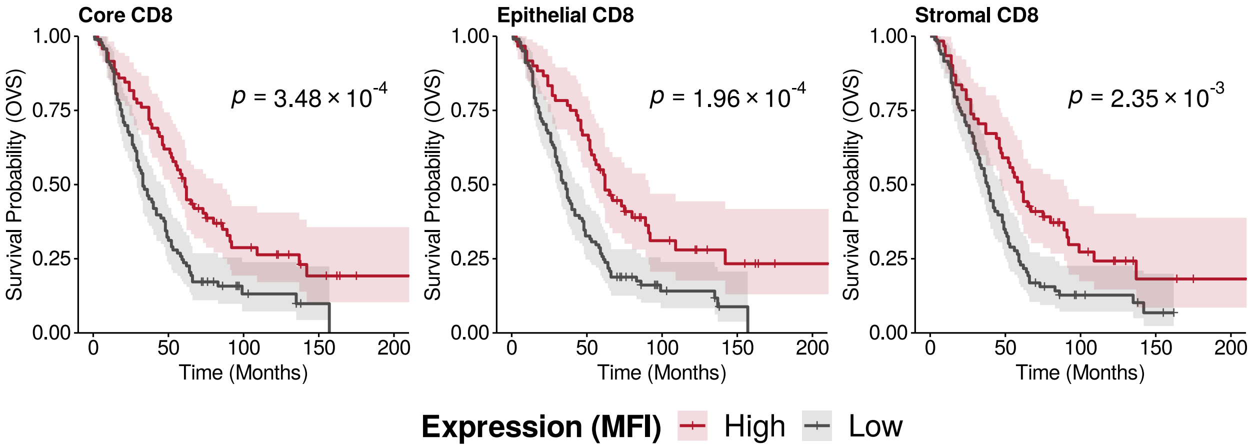

Supplementary Fig. S5: CD8 expression and overall patient survival (OS). Kaplan-Meier survival plots display survival outcomes for dichotomized CD8 expression groups (low vs. high). P-values were computed using the log-rank test.

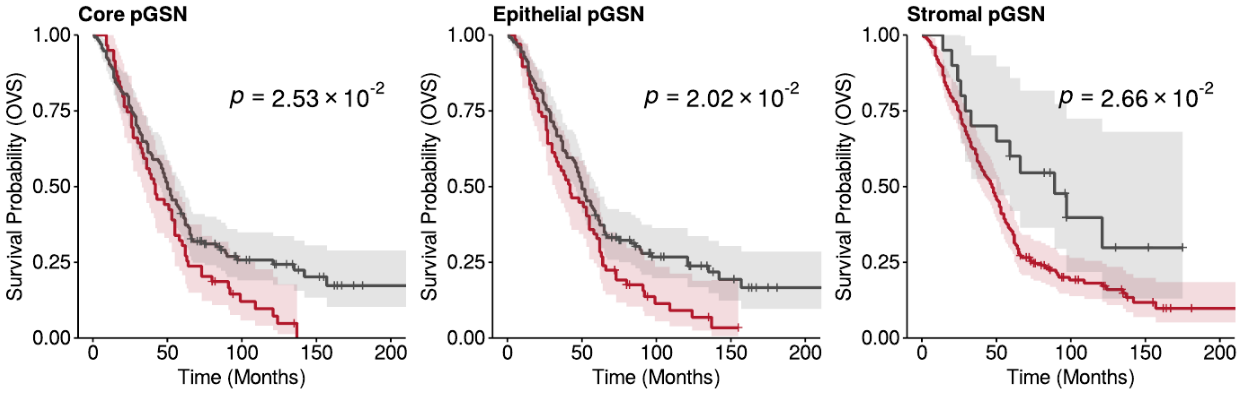

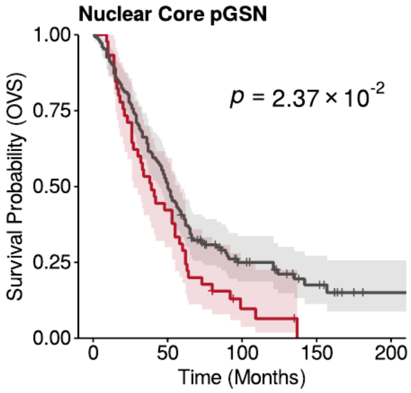

Supplementary Fig. S6: pGSN expression and overall patient survival (OS). Kaplan-Meier survival plots display survival outcomes for dichotomized pGSN expression groups (low vs. high). P-values were computed using the log-rank test.

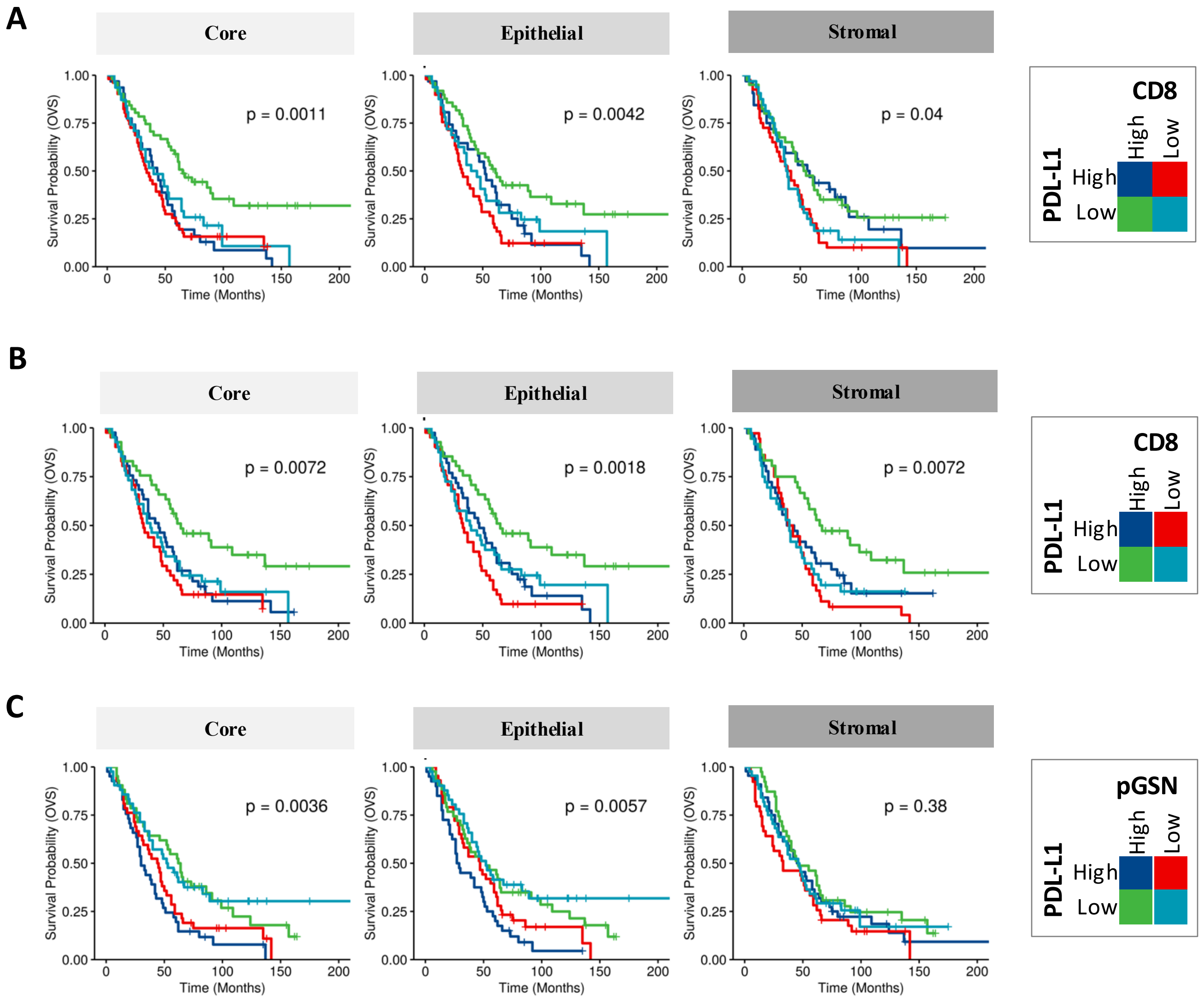

Supplementary Fig. S7: Increased nuclear PD-L1 and pGSN hinder the anti-tumor benefits of infiltrated CD8+ T cells in the OVCA microenvironment and associated with overall survival (OVS). KM plot in (A) shows PD-L1 expression in combination with infiltrated CD8+ T cells in the core, epithelial and stromal compartments. (B) Epithelial nuclear PD-L1 expression, combined with CD8+ T cell presence in the core, epithelium, and stroma. (C) PD-L1 in combination with pGSN expression in the core, epithelium, and stroma. For the analysis, samples were classified into four groups based on the expression levels of two markers. Each marker was dichotomized into high and low expression, resulting in four groups: high-high, high-low, low-high, and low-low. Kaplan–Meier survival plots depict disease-free survival probabilities across these groups for the respective markers. Survival distributions were compared using the log-rank test.

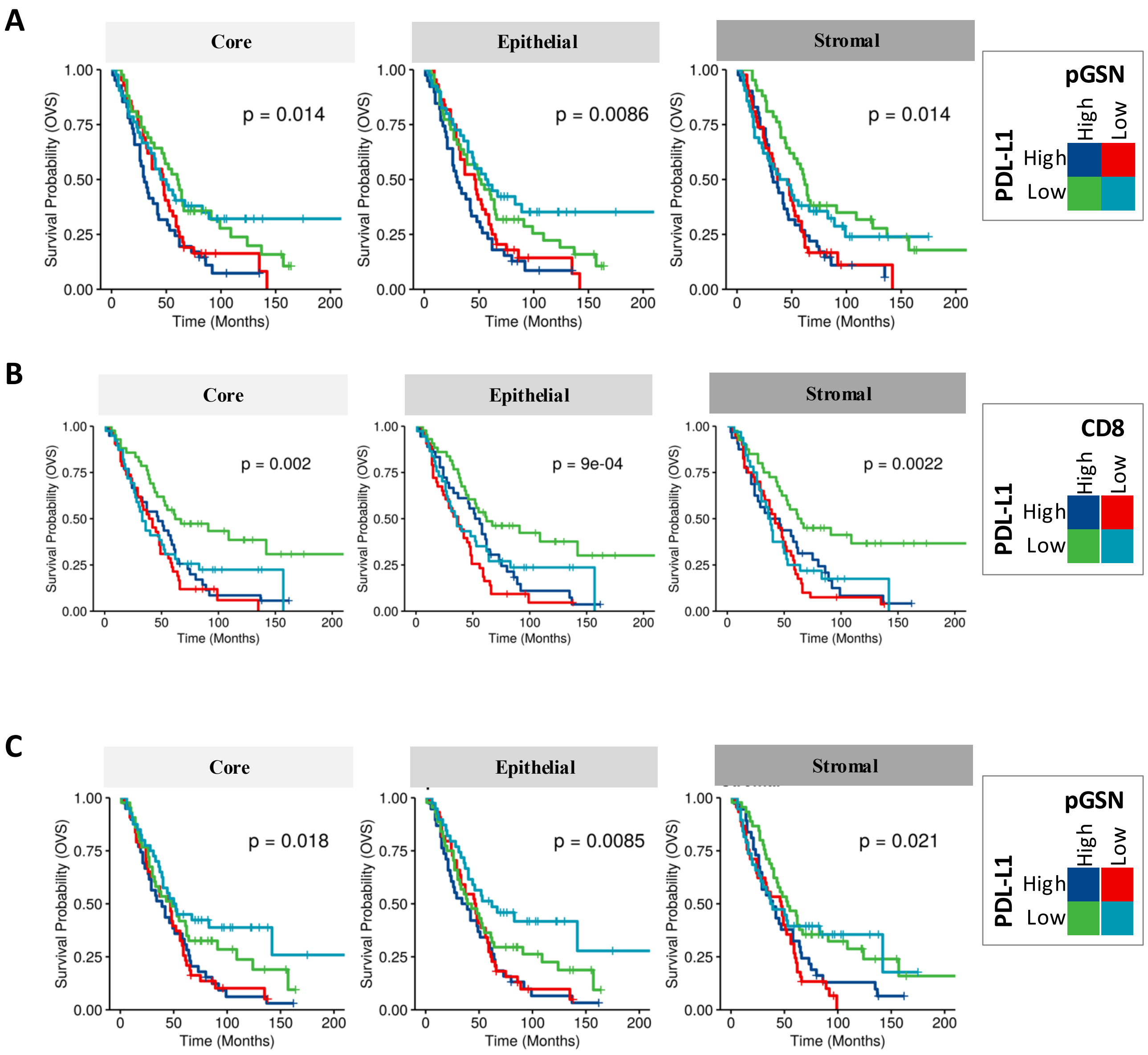

Supplementary Fig. S8: Kaplan–Meier survival plots for marker expression groups and overall survival (OS). Kaplan-Meier survival plots for (A) Epithelial nuclear PD-L1 expression, in combination with pGSN expression in the core, epithelium, and stroma. (B) Stromal nuclear PD-L1 expression, combined with CD8+ T cell presence in the core, epithelium, and stroma. (C) Stromal nuclear PD-L1 expression, in combination with pGSN expression in the core, epithelium, and stroma. For each combination core, epithelium, and stroma compartments were analyzed. Survival distributions were compared using the log-rank test.

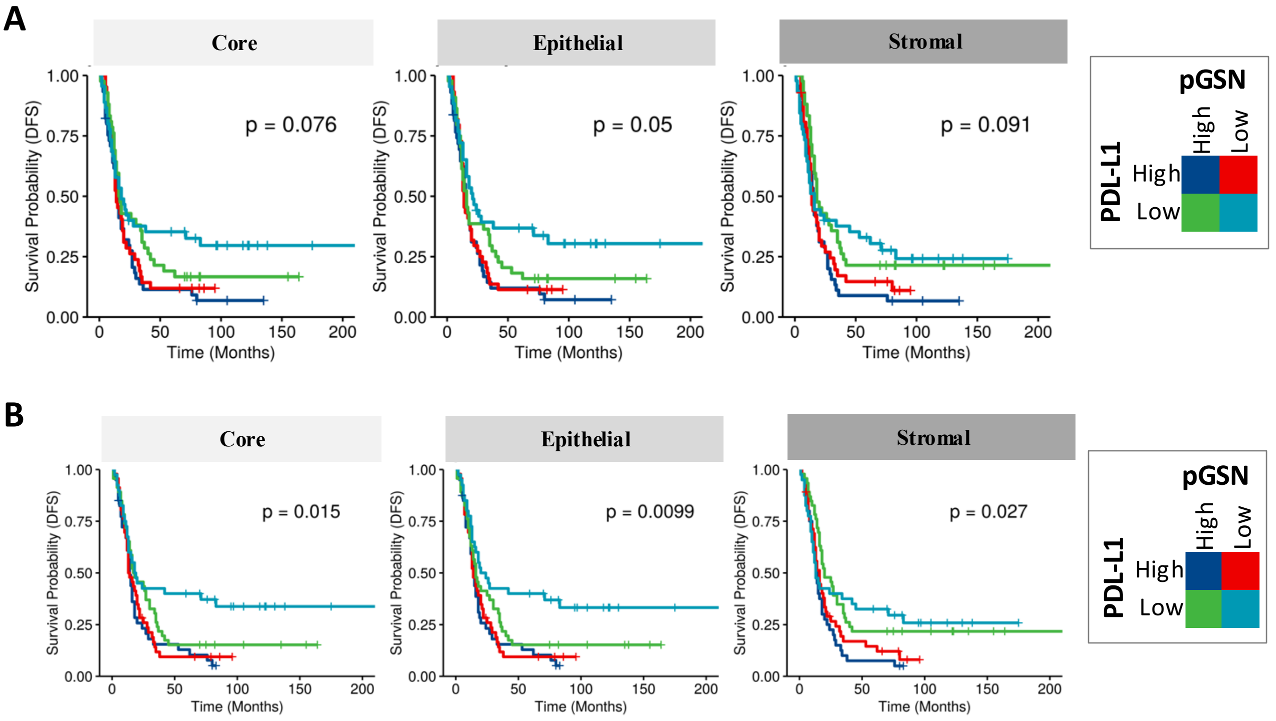

Supplementary Fig. S9: Kaplan–Meier survival plots for marker expression groups and DFS. Kaplan-Meier survival plots for (A) Epithelial nuclear PD-L1 expression, in combination with pGSN expression in the core, epithelium, and stroma. (B) Stromal nuclear PD-L1 expression, in combination with pGSN expression in the core, epithelium, and stroma. For each combination core, epithelium, and stroma compartments were analyzed. Survival distributions were compared using the log-rank test.

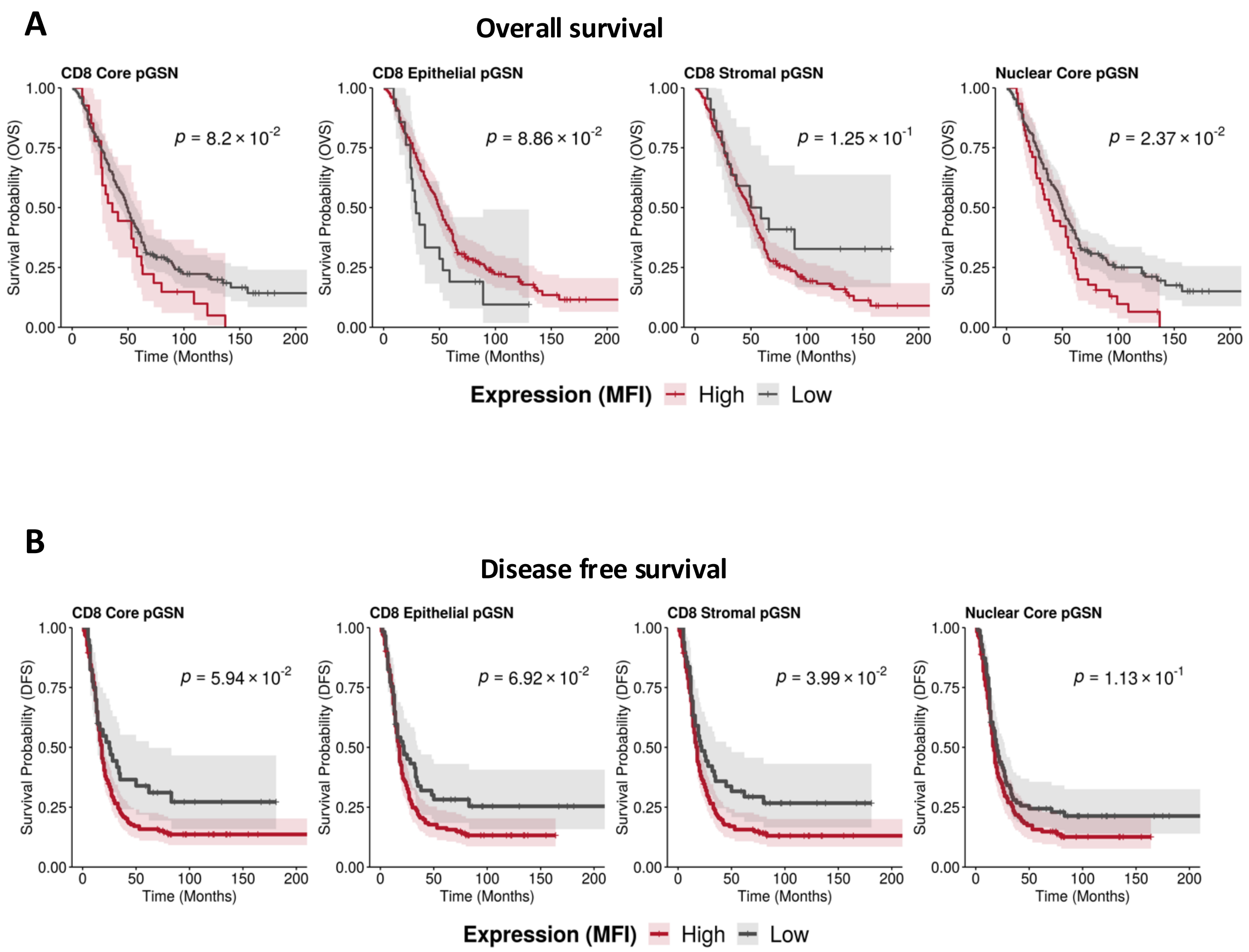

Supplementary Fig. S10: Increased pGSN localization in CD8 T cells is associated with poor patient survival. pGSN localization in CD8 T cells in both the epithelial and stroma as well as nulcear pGSN were correlated with DFS and OS. Kaplan–Meier survival curves with dichotomized pGSN expression (low and high groups; optimal cut-offs) and log-rank test were used to compare the survival distributions between the groups.

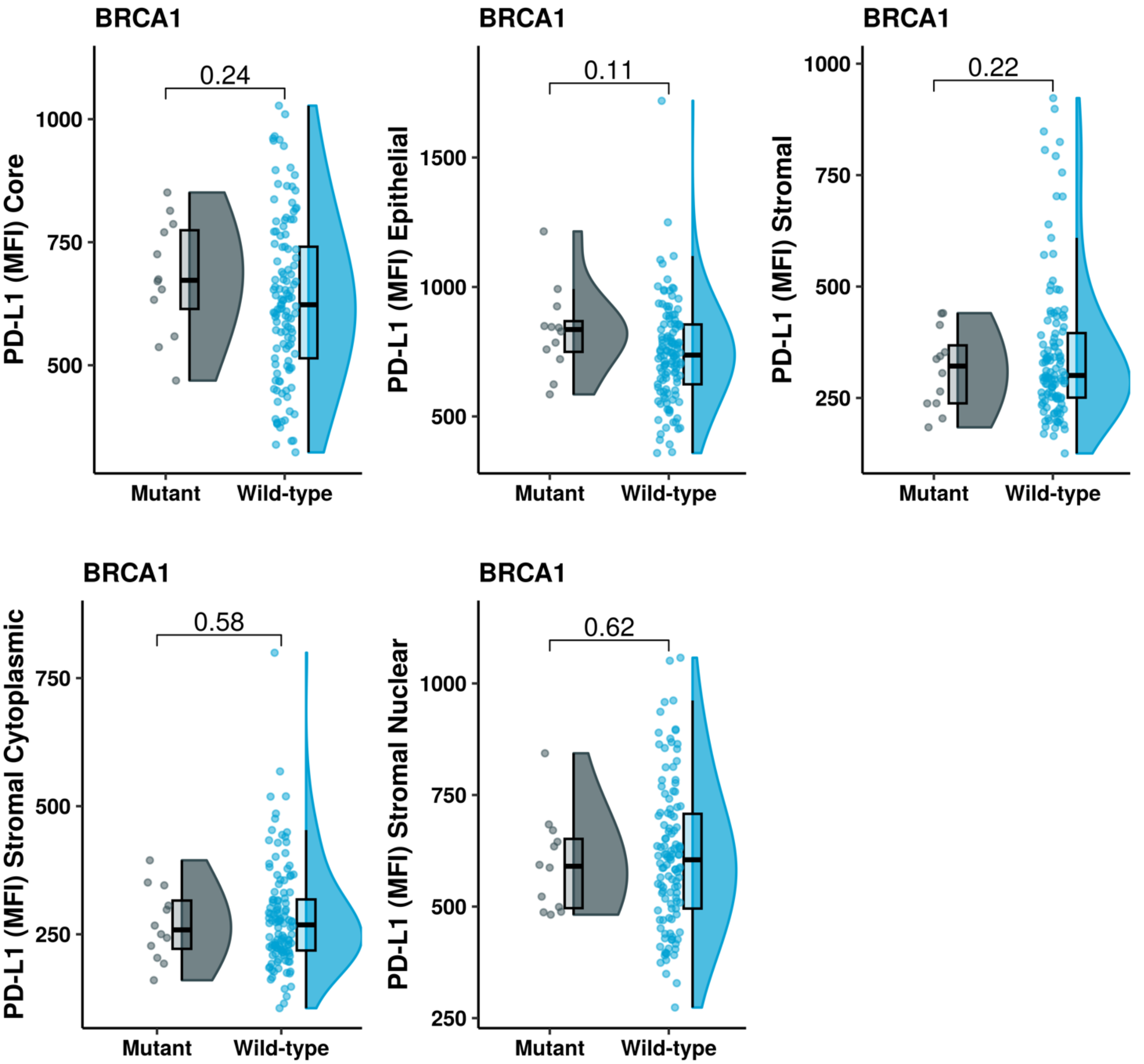

Supplementary Figure S11: Raincloud plots shows PD-L1 Core, Epithelial, Stromal, Stromal Cytoplasmic, and Stromal Nuclear levels as stratified by BRCA1 mutation status. The median difference between the two groups was compared using Student's t-test and p-value is indicated on the top.

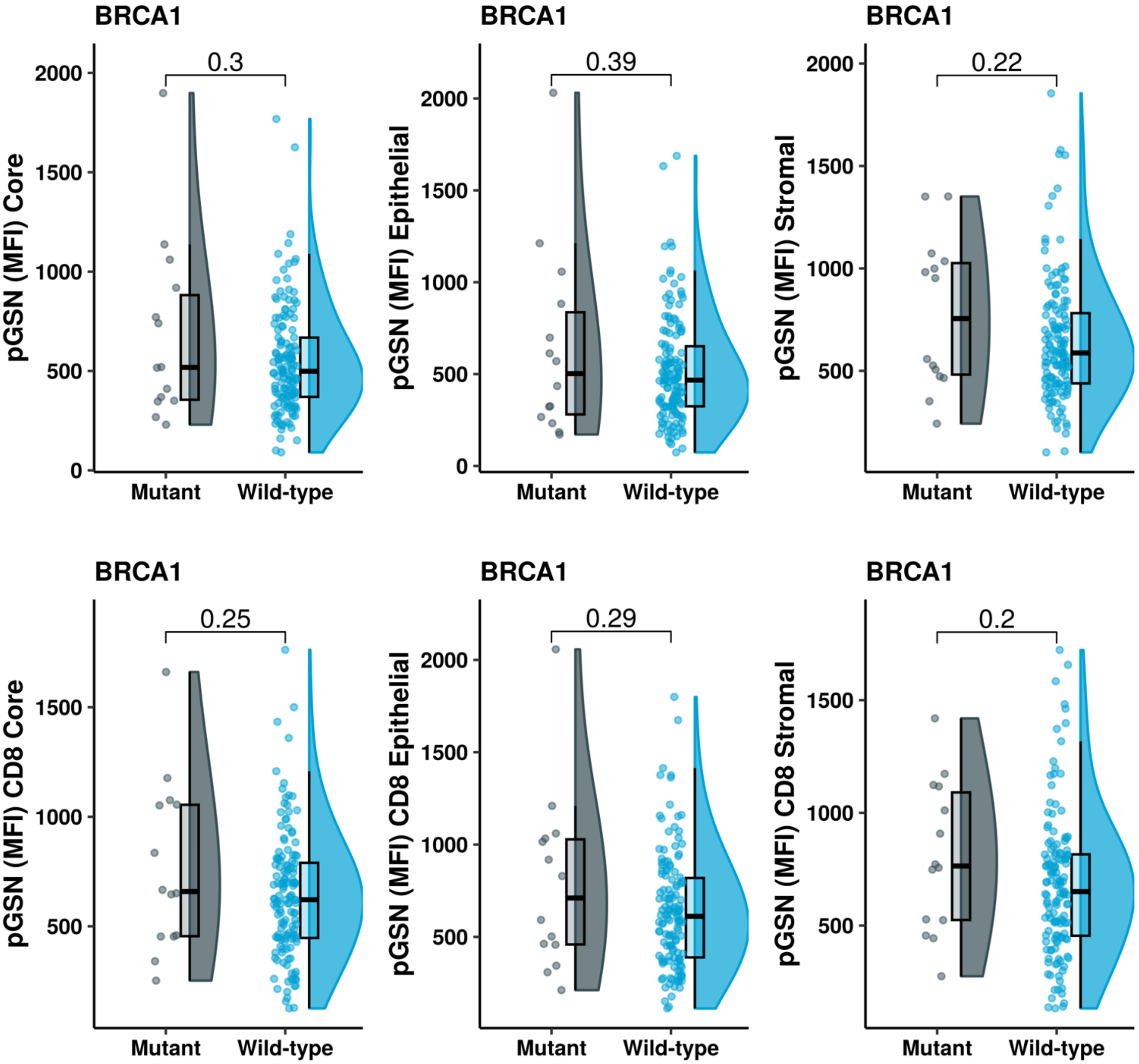

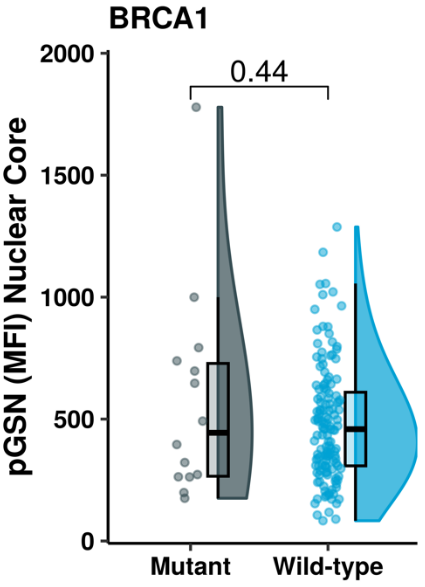

Supplementary Figure S12: Raincloud plots shows pGSN Core, Epithelial, Stromal, CD8 Core, CD8 Epithelial, CD8 Stromal, and Nuclear Core levels as stratified by BRCA1 mutation status. The median difference between the two groups was compared using Student's t-test and p-value is indicated on the top.

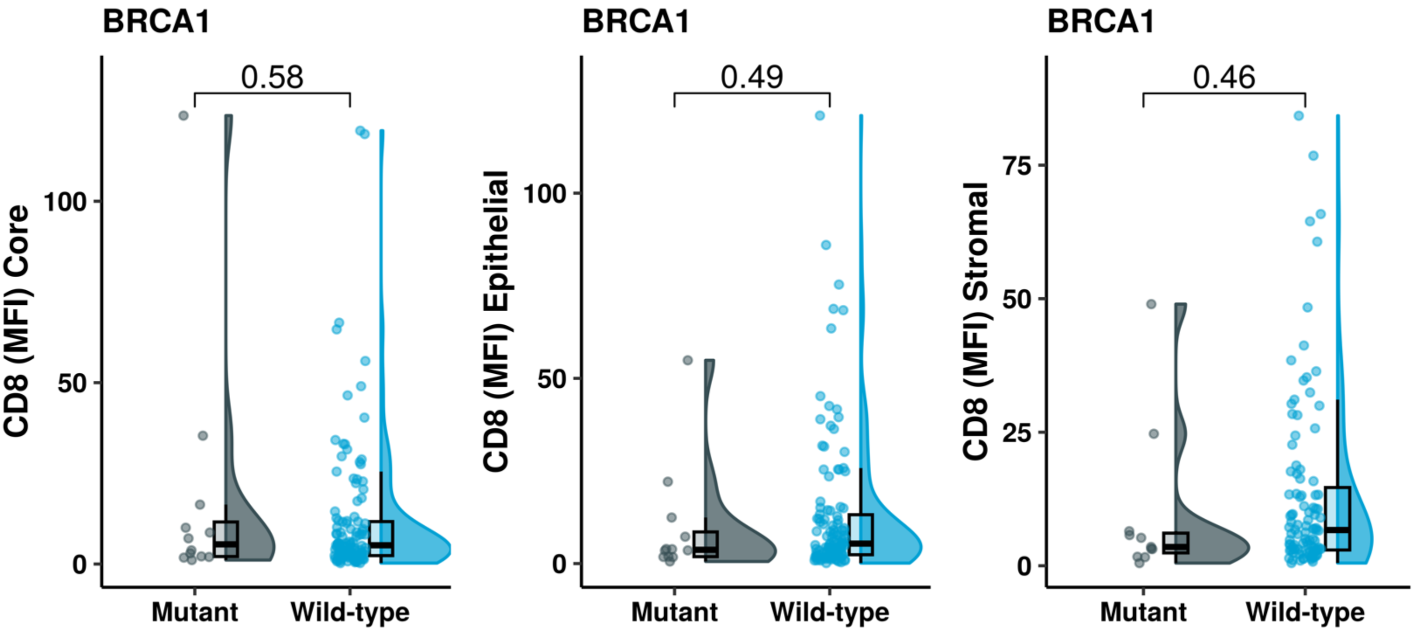

Supplementary Figure S13: Raincloud plots shows CD8 Core, Epithelial, and Stromal levels as stratified by BRCA1 mutation status. The median difference between the two groups was compared using Student's t-test and p-value is indicated on the top.

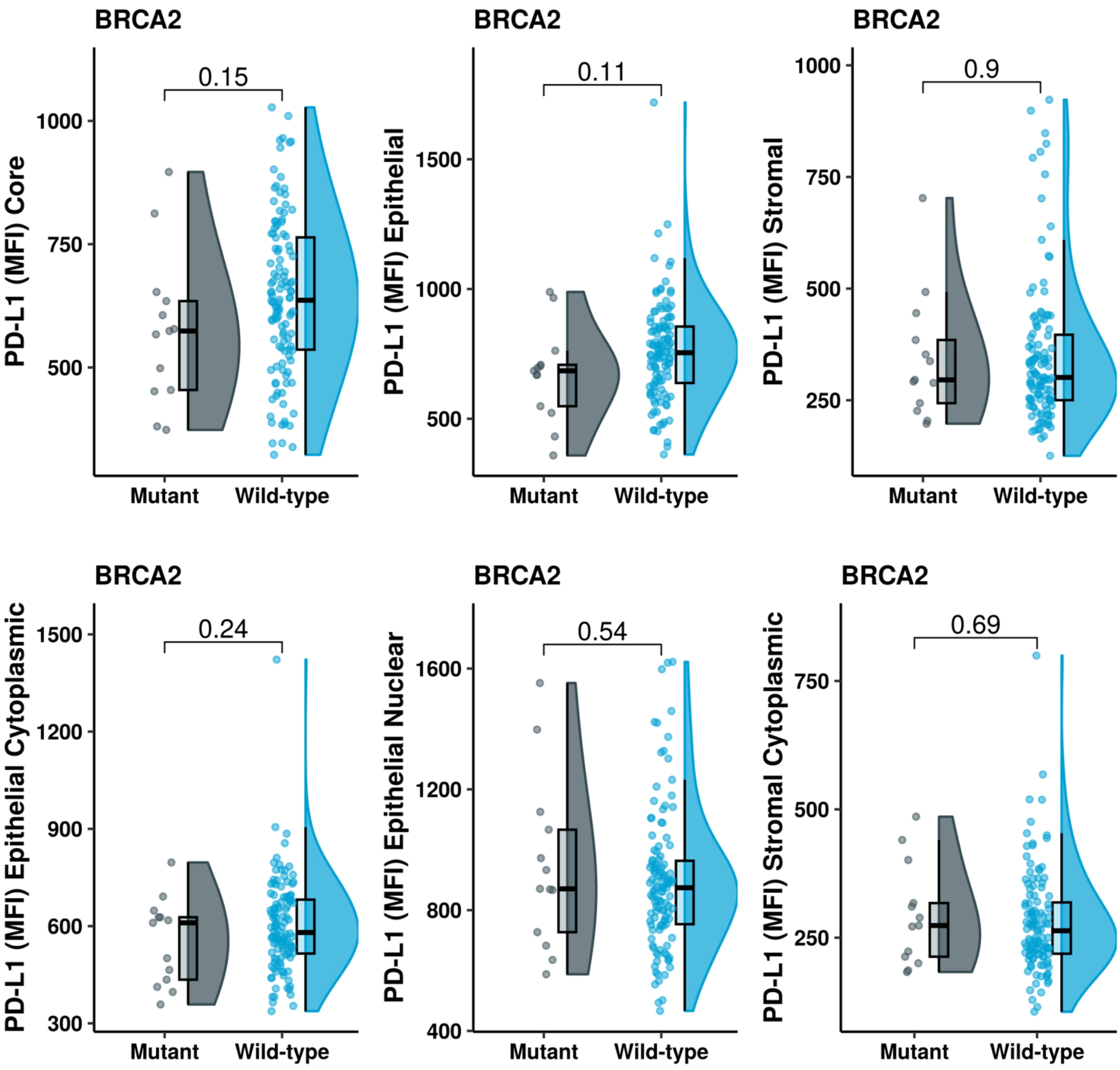

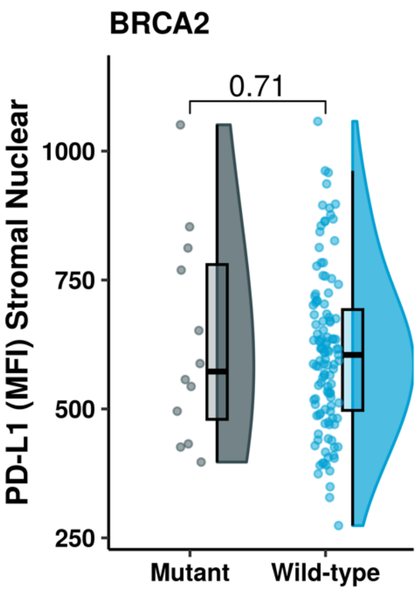

Supplementary Figure S14: Raincloud plots shows PD-L1 Core, Epithelial, Stromal, Epithelial Cytoplasmic, Epithelial Nuclear, Stromal Cytoplasmic, Stromal Nuclear levels as stratified by BRCA2 mutation status. The median difference between the two groups was compared using Student's t-test and p-value is indicated on the top.

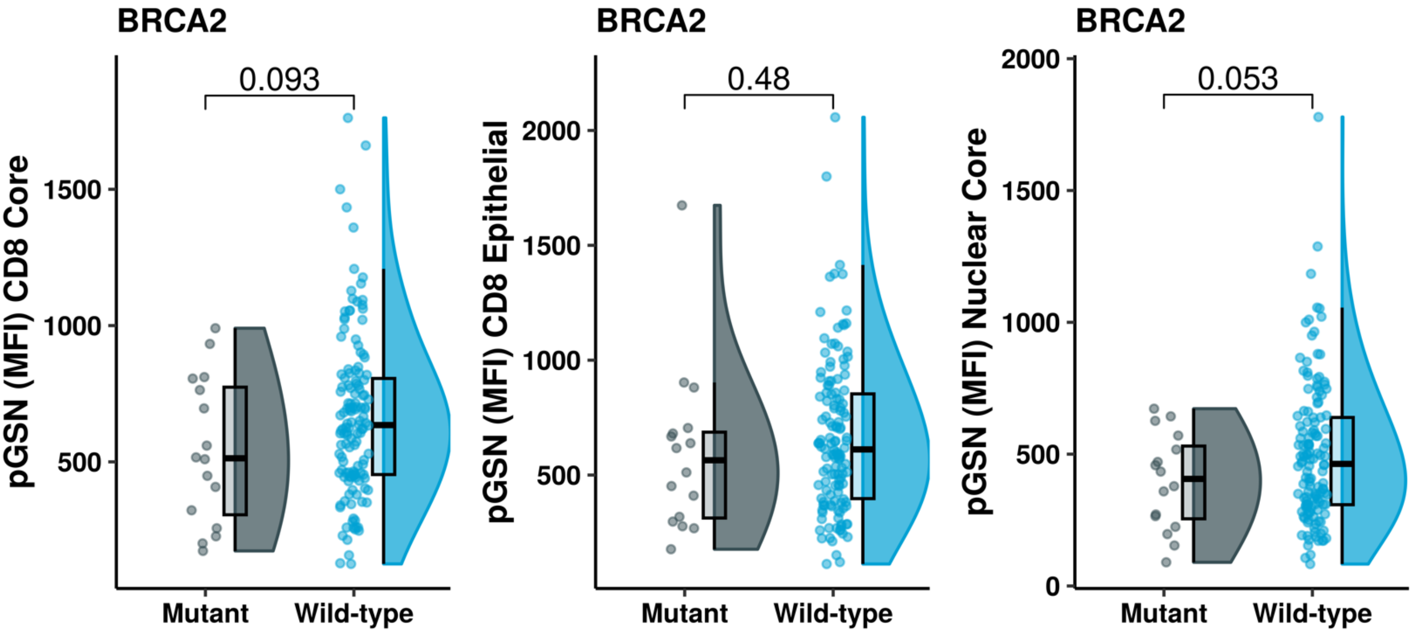

Supplementary Figure S15: Raincloud plots shows pGSN Core, Epithelial, and Stromal levels as stratified by BRCA2 mutation status. The median difference between the two groups was compared using Student's t-test and p-value is indicated on the top.

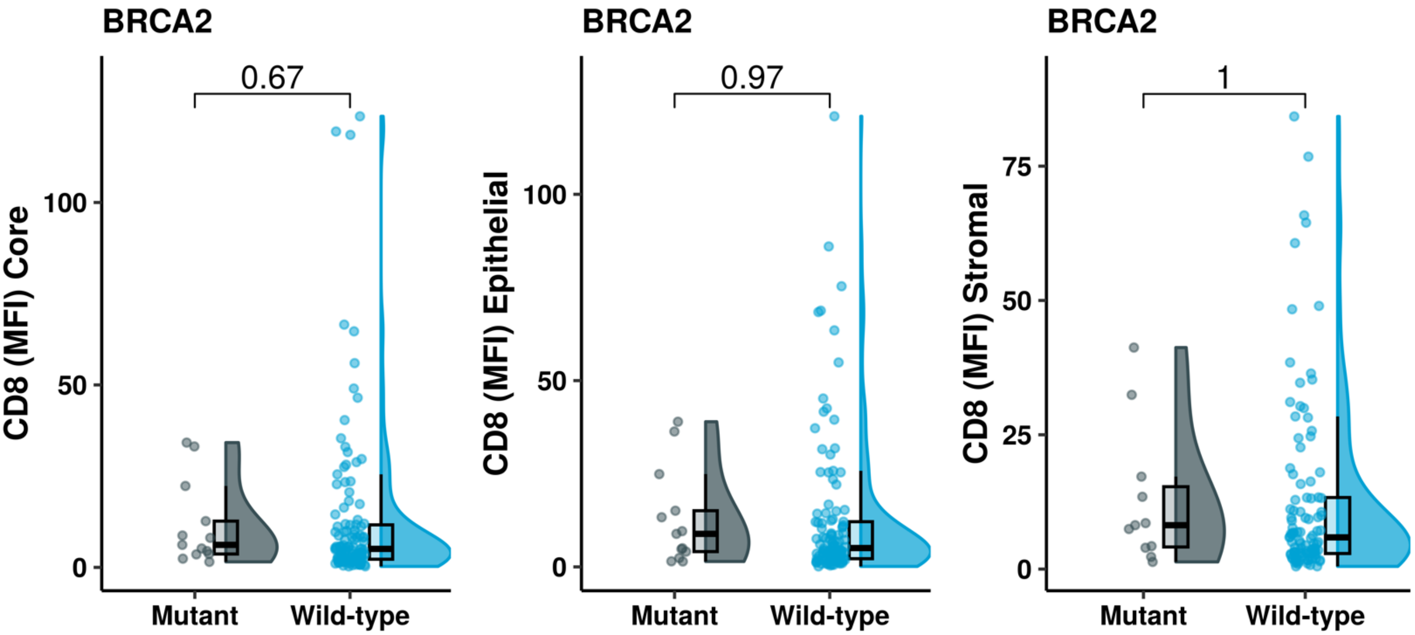

Supplementary Figure S16: Raincloud plots shows CD8 Core, Epithelial, and Stromal levels as stratified by BRCA2 mutation status. The median difference between the two groups was compared using Student's t-test and p-value is indicated on the top.

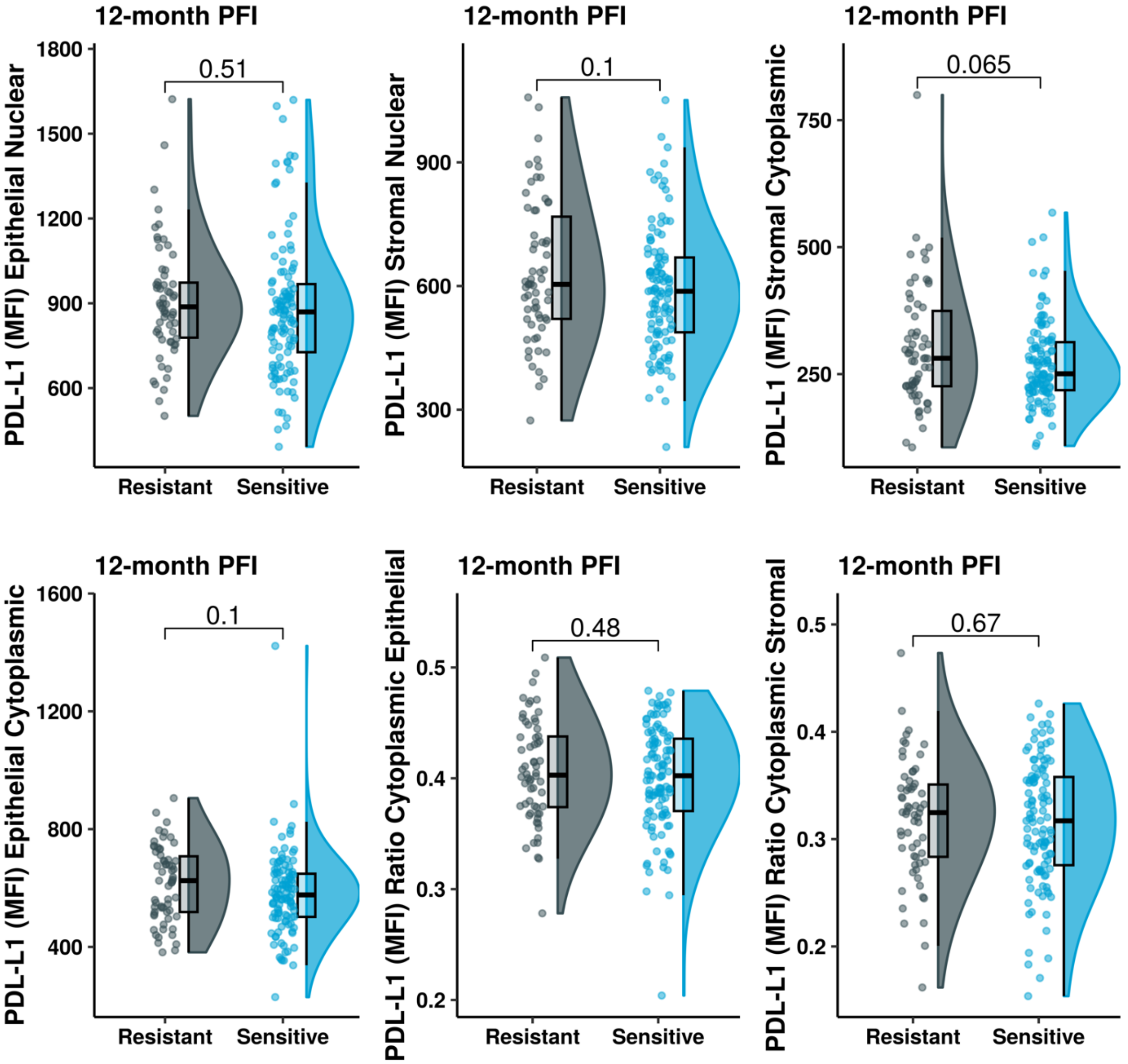

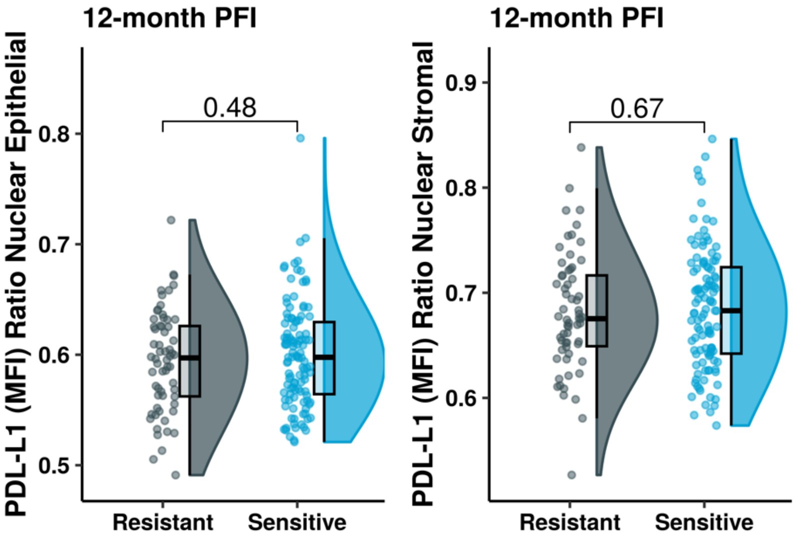

Supplementary Figure S17: Raincloud plots shows PD-L1 Epithelial Nuclear, Stromal Nuclear, Stromal Cytoplasmic, Epithelial Cytoplasmic, Cytoplasmic Epithelial Ratio, Cytoplasmic Stromal Ratio, Nuclear Epithelial Ratio, and Nuclear Stromal Ratio levels as stratified by 12-month PFI status. The median difference between the two groups was compared using Student's t-test and p-value is indicated on the top.

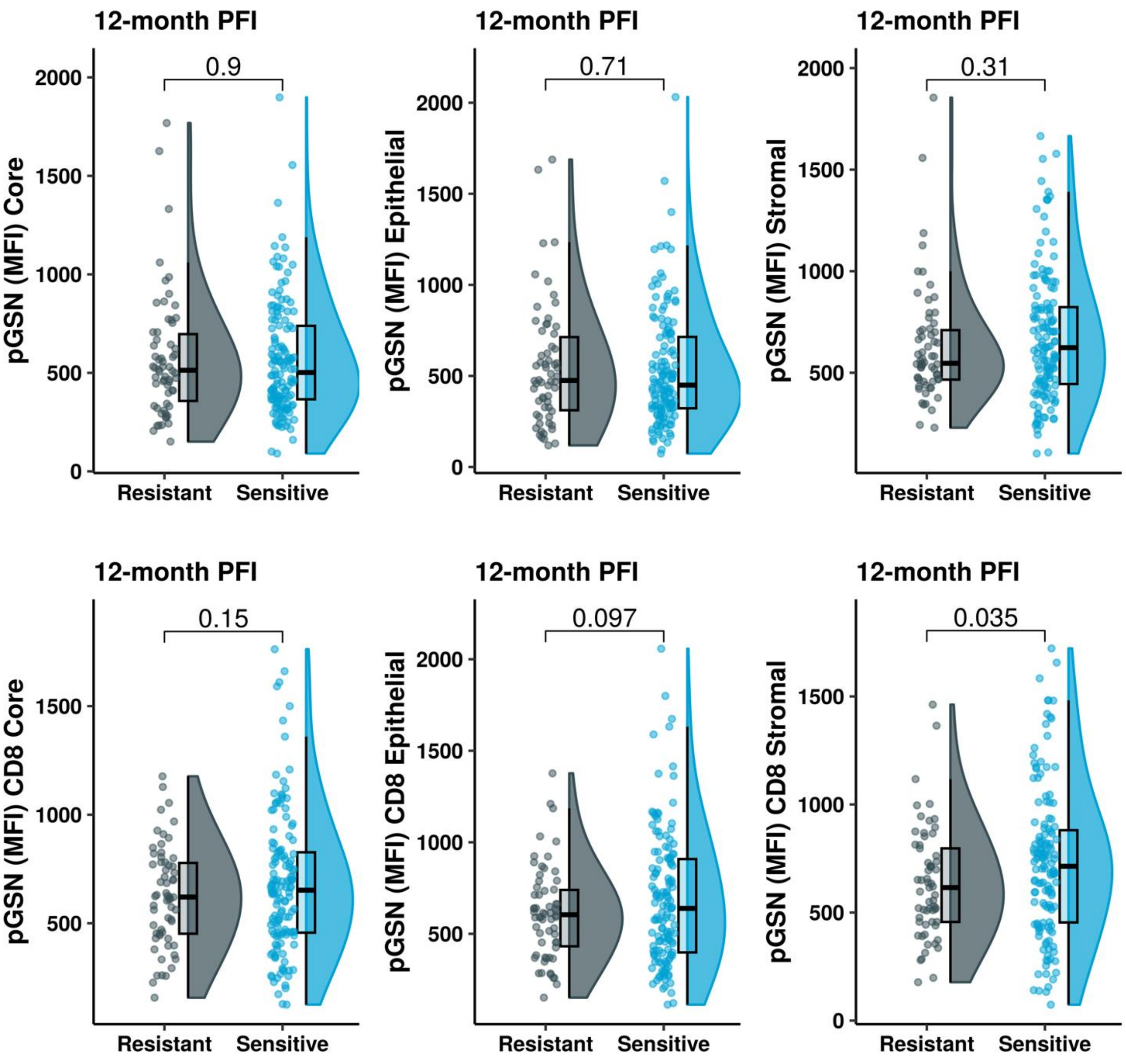

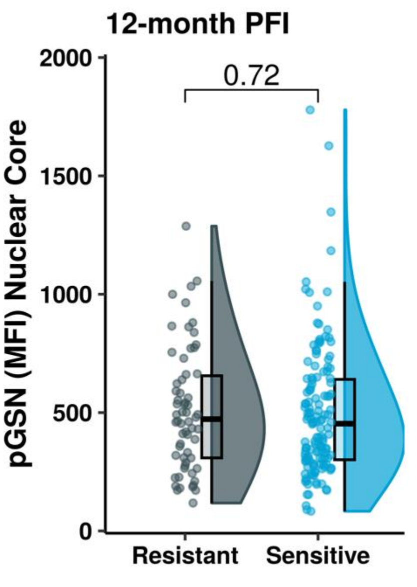

Supplementary Figure S18: Raincloud plots shows pGSN Core, Epithelial, Stromal, CD8 Core, CD8 Epithelial, CD8 Stromal, and Nuclear Core levels as stratified by 12-month PFI status. The median difference between the two groups was compared using Student's t-test and p-value is indicated on the top.

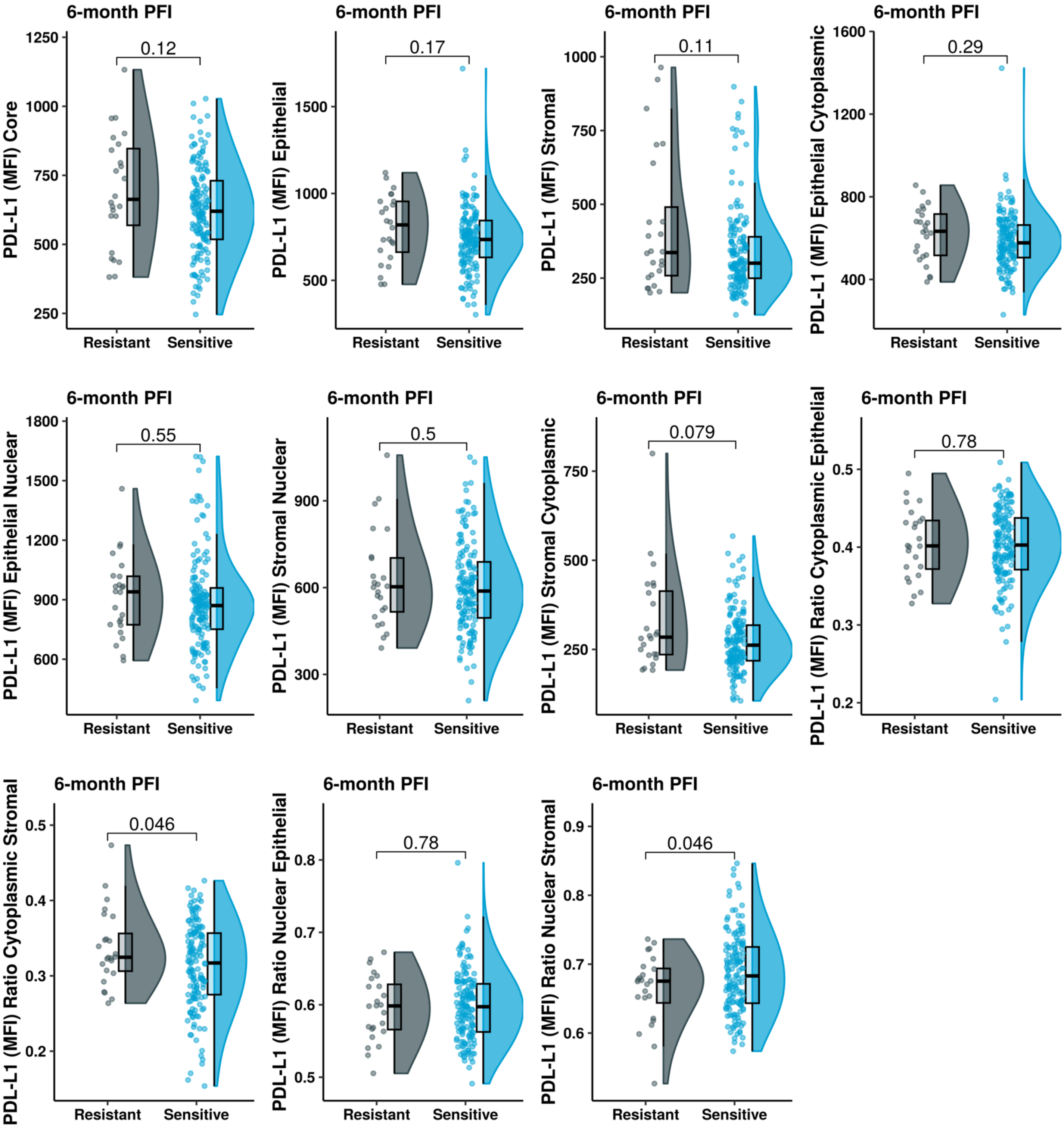

Supplementary Figure S19: Raincloud plots shows PD-L1 Core, Epithelial, Stromal, Epithelial Cytoplasmic, Epithelial Nuclear, Stromal Nuclear, Stromal Cytoplasmic, Ratio Cytoplasmic Epithelia, Ratio Cytoplasmic Stromal, Ratio Nuclear Epithelial, Ratio Nuclear Stromal levels as stratified by 6-month PFI status. The median difference between the two groups was compared using Student's t-test and p-value is indicated on the top.

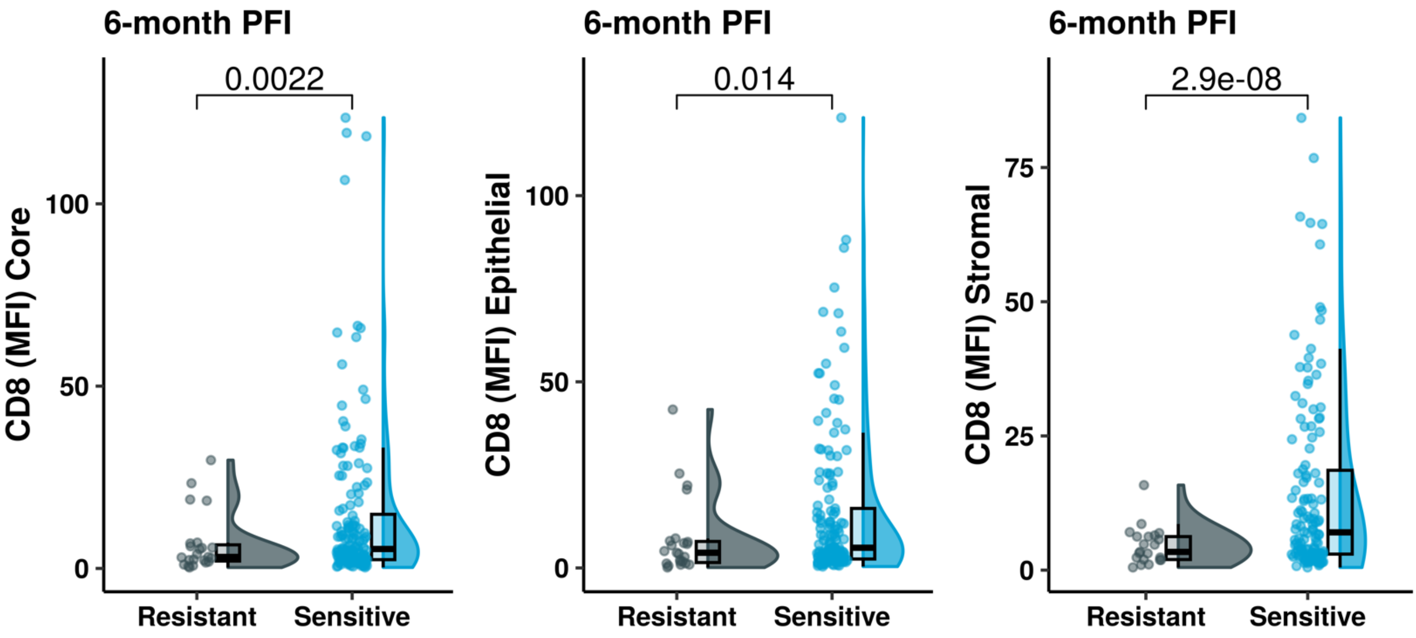

Supplementary Figure S20: Raincloud plots shows CD8 Core, Epithelial, and Stromal levels as stratified by 6-month PFI status. The median difference between the two groups was compared using Student's t-test and p-value is indicated on the top.

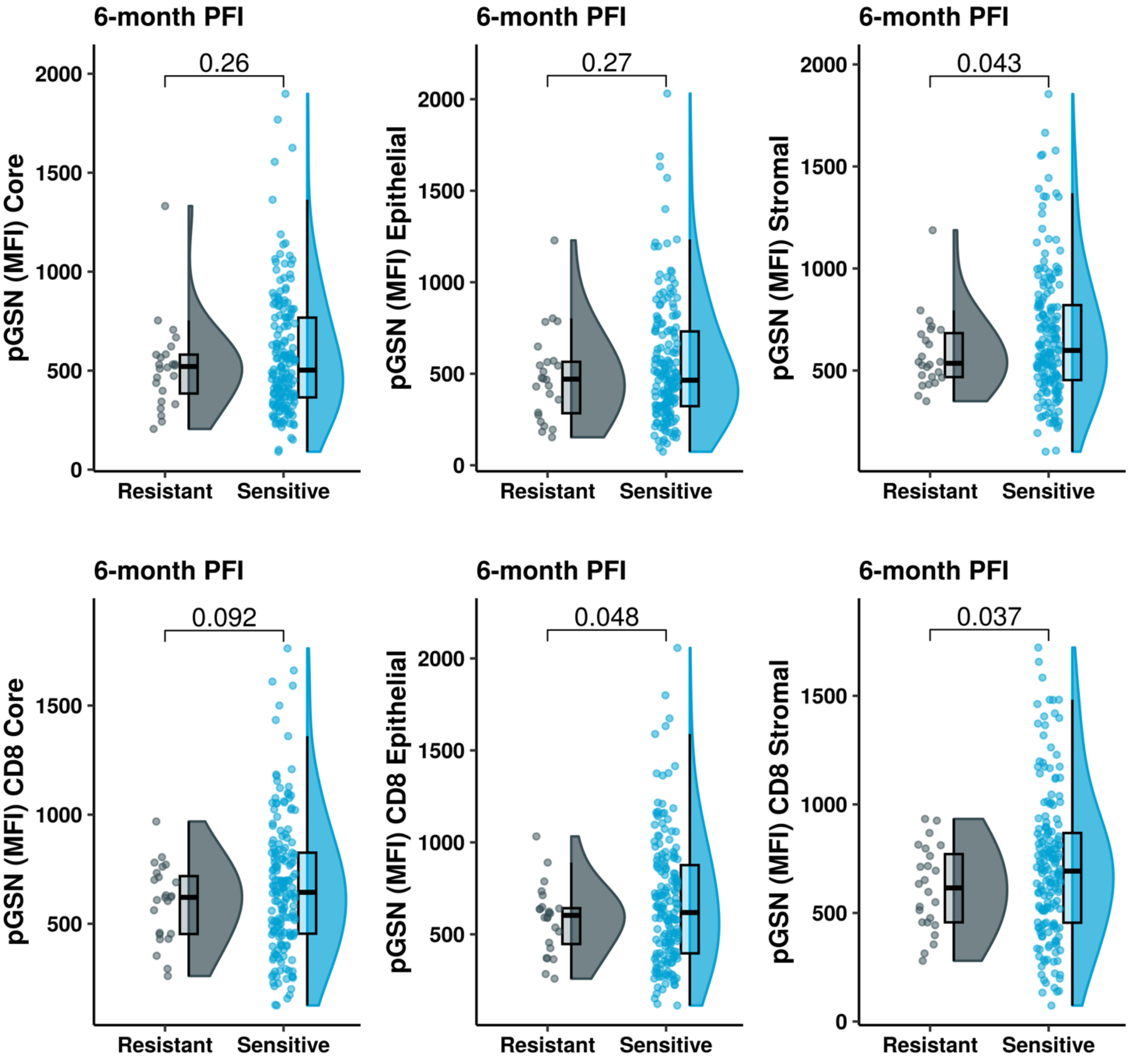

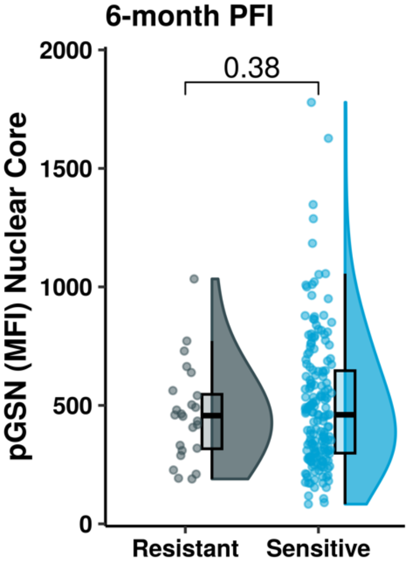

Supplementary Figure S21: Raincloud plots shows pGSN in the Core, Epithelial, Stromal compartments, CD8 Core, CD8 Epithelial, CD8 Stromal, and Nuclear Core levels as stratified by 6-month PFI status. The median difference between the two groups was compared using Student's t-test and p-value is indicated on the top.

Supplementary Tables

Supplementary Table S1: Univariate Cox proportional hazards model-based analysis of overall survival

Predictor Variable	HR (95% CI for HR)	P-value
**Age**	1.017 (1.001-1.034)	0.0396 *
**Menopausal status**	1.241 (0.6825-2.258)	0.5
**FIGO Stage 2**	1.382 (0.4799-3.979)	0.549
**FIGO Stage 3**	2.826 (1.1547-6.918)	0.0229 *
**FIGO Stage 4**	4.240 (1.5939-11.278)	0.0038 **
**CA125**	1 (0.9999-1)	0.725
**RD Dichotomized**	1.911 (1.371-2.665)	0.000133 ***
**PDL1 Core**	1.366 (1.145-1.631)	6e-04 ***
**PDL1 Epithelial**	1.285 (1.112-1.487)	7e-04 ***
**PDL1 Stromal**	1.261 (1.068-1.488)	0.00624 **
**Epithelial Nuclear PDL1**	1.273 (1.089-1.488)	0.00246 **
**Epithelial Cytoplasmic PDL1**	1.196 (1.039-1.377)	0.0125 *
**Stromal Nuclear PDL1**	1.348 (1.145-1.588)	0.000349 ***
**Stromal Cytoplasmic PDL1**	1.189 (1.017-1.391)	0.0298 *
**CD8 Core**	0.7914 (0.6325-0.9901)	0.0407 *
**CD8 Epithelial**	0.7912 (0.6373-0.9825)	0.034 *
**CD8 Stroma**	0.7992 (0.6419-0.9951)	0.0451 *
**BRCA1 Status**	0.811 (0.4359-1.509)	0.508
**BRCA2 Status**	0.5361 (0.2804-1.025)	0.0594
**pGSN Core**	1.187 (1.009-1.398)	0.039 *
**pGSN Epithelial**	1.201 (1.021-1.414)	0.0275 *
**pGSN Stroma**	1.054 (0.8991-1.236)	0.515
**pGSN in CD8 Core**	1.125 (0.9574-1.323)	0.152
**pGSN in CD8 Epithelial**	1.078 (0.9203-1.264)	0.35
**pGSN in CD8 Stroma**	1.052 (0.8932-1.239)	0.544
**pGSN Nuclei Core**	1.181 (1.005-1.388)	0.0438 *

Supplementary Table S2: Univariate Cox proportional hazards model-based analysis of disease-free survival

Predictor Variable	HR (95% CI for HR)	P-value
**Age**	1.005 (0.9896-1.02)	0.539
**Menopausal status**	0.9508 (0.5451-1.659)	0.859
**FIGO Stage 2**	2.180 (0.7768-6.119)	0.138783
**FIGO Stage 3**	4.634 (1.8912-11.355)	0.000798 ***
**FIGO Stage 4**	8.314 (3.1123-22.208)	2.39e-05 ***
**CA125**	1 (0.9999-1)	0.984
**RD Dichotomized**	2.6 (1.879-3.599)	8.16e-09 ***
**PDL1 Core**	1.373 (1.158-1.628)	0.000268 ***
**PDL1 Epithelial**	1.256 (1.091-1.445)	0.00146 **
**PDL1 Stromal**	1.376 (1.161-1.632)	0.000235 ***
**Epithelial Nuclear PDL1**	1.223 (1.049-1.426)	0.00996 **
**Epithelial Cytoplasmic PDL1**	1.181 (1.028-1.357)	0.0189 *
**Stromal Nuclear PDL1**	1.363 (1.153-1.612)	0.000292 ***
**Stromal Cytoplasmic PDL1**	1.259 (1.069-1.481)	0.00562 **
**CD8 Core**	0.7316 (0.5892-0.9084)	0.00465 **
**CD8 Epithelial**	0.7328 (0.5946-0.9031)	0.00355 **
**CD8 Stromal**	0.728 (0.5888-0.9002)	0.00338 **
**BRCA1 Status**	1.005 (0.5657-1.787)	0.985
**BRCA2 Status**	0.4896 (0.2563-0.9352)	0.0306 *
**pGSN Core**	1.096 (0.9443-1.272)	0.228
**pGSN Epithelial**	1.089 (0.9374-1.266)	0.265
**pGSN Stroma**	1.064 (0.9199-1.232)	0.401
**pGSN in CD8 Core**	1.041 (0.9009-1.203)	0.585
**pGSN in CD8 Epithelial**	1.02 (0.8811-0.8811)	0.792
**pGSN in CD8 Stroma**	1.01 (0.8724-1.169)	0.894
**pGSN Nuclei Core**	1.084 (0.9373-1.254)	0.276

Supplementary Table S3: Cox proportional hazards model-based multivariable analyses of overall survival

		HR	*P* value
**Age**		0.99 *(0.951 - 1.04)*	*0.726*
**Menopause status**	*0*	reference	
	1	2.46 *(0.930 - 6.50)*	*0.07*
**FIGO**	*1*	reference	
	2	16.63 *(2.658 - 104.08)*	*0.003 ***
	3	11.55 *(2.174 - 61.38)*	*0.004 ***
	4	44.17 *(6.776 - 287.97)*	*<0.001 ****
**CA125**		0.89 *(0.713 - 1.11)*	*0.3*
**RD Dichotomized**	*0*	reference	
	1	1.95 *(1.021 - 3.73)*	*0.043 **
**Core PDL1**		2.14 *(1.083 - 4.24)*	*0.029 **
**Epithelial PDL1**		1.93 *(0.639 - 5.81)*	*0.244*
**Stromal PDL1**		1.02 *(0.451 - 2.30)*	*0.964*
**Epithelial Nuclear PDL1**		1.00 *(0.144 - 6.95)*	*0.999*
**Epithelial Cytoplasmic PDL1**		0.47 *(0.081 - 2.77)*	*0.407*
**Stromal Nuclear PDL1**		0.26 *(0.062 - 1.06)*	*0.06*
**Stromal Cytoplasmic PDL1**		3.58 *(0.785 - 16.32)*	*0.099*
**Core CD8**		2.12 *(0.580 - 7.73)*	*0.257*
**Epithelial CD8**		0.55 *(0.198 - 1.55)*	*0.26*
**Stromal CD8**		1.00 *(0.508 - 1.98)*	*0.992*
**Core pGSN**		0.89 *(0.186 - 4.31)*	*0.89*
**Epithelial pGSN**		2.28 *(0.667 - 7.80)*	*0.188*
**Stromal pGSN**		1.01 *(0.625 - 1.63)*	*0.97*
**pGSN Core Nuclear**		0.63 *(0.210 - 1.88)*	*0.404*
**Epithelial Cytoplasmic Ratio PDL1**		1.31 *(0.243 - 7.04)*	*0.755*
**Stromal Cytoplasmic Ratio PDL1**		0.20 *(0.044 - 0.92)*	*0.039 **
**BRCA1/2 Status**	*Wild-type*	reference	
	Mutant	0.91 *(0.453 - 1.83)*	*0.795*

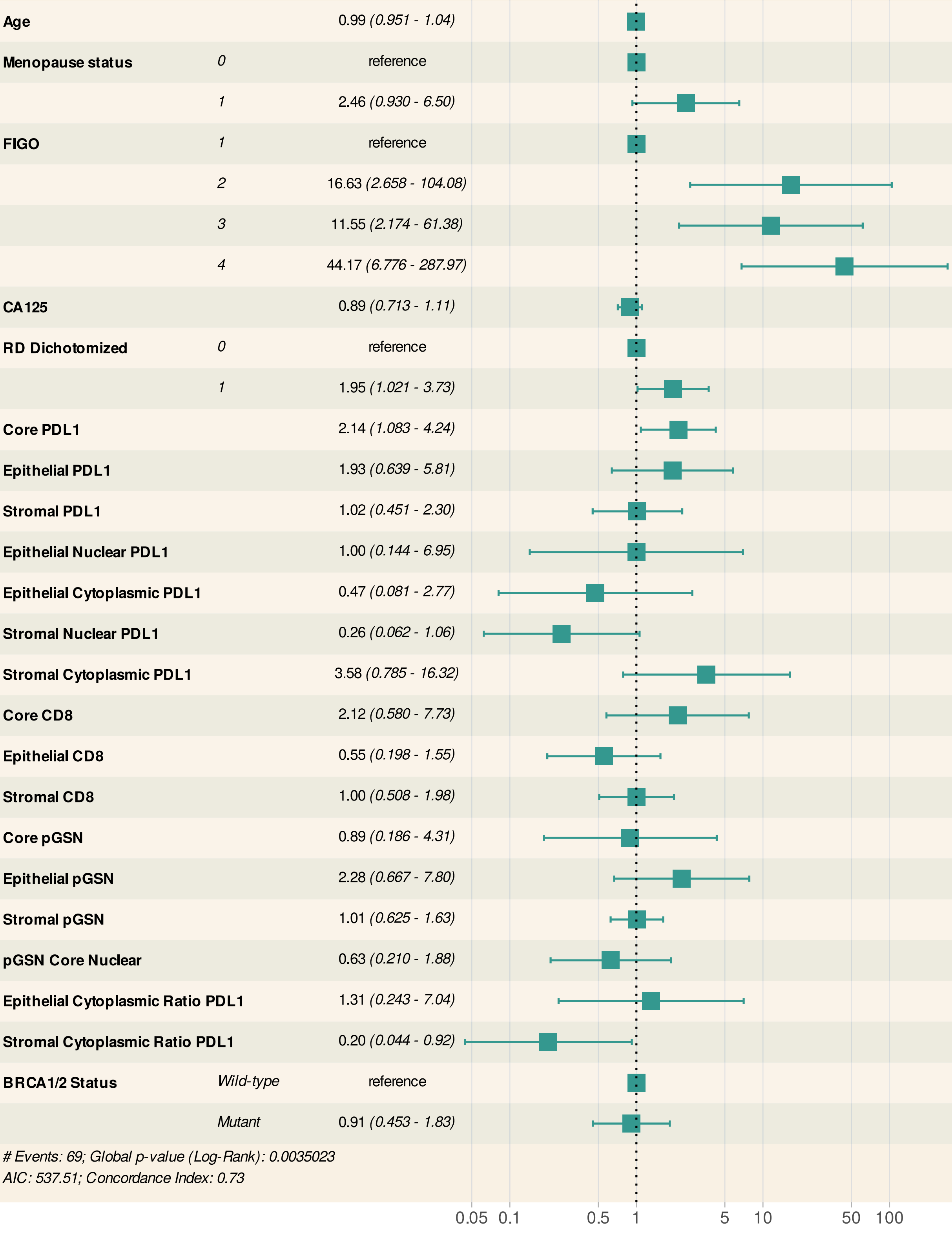

# Events: 69; Global p-value (Log-Rank): 0.0035023
AIC: 537.51; Concordance Index: 0.73
